# Replacement of Isoleucine and Leucine by their Keto Acids leads to increased formation of α‐Hydroxy Acids in Chinese Hamster Ovary cells

**DOI:** 10.1002/biot.70041

**Published:** 2025-06-09

**Authors:** Philipp Reifenberg, Lara Rosenberger, Maxime Le Mignon, Aline Zimmer

**Affiliations:** ^1^ Merck Life Science KGaA Upstream R&D Darmstadt Germany; ^2^ Institute for Organic Chemistry and Biochemistry Technische Universität Darmstadt Darmstadt Germany; ^3^ Merck KGaA Darmstadt Germany

**Keywords:** α‐hydroxy acids, α‐keto acids, branched‐chain amino acids, isoleucine, leucine, valine

## Abstract

Keto isoleucine and keto leucine are bioavailable amino acid precursors in CHO cells producing biotherapeutics. Following their supplementation in a fed‐batch culture with CHOK1 GS cells, quantitation of metabolites derived from the three branched‐chain keto acids revealed a decrease of their decarboxylation products, but an accumulation of their α‐hydroxy acids. The latter had minor effects on cell growth and productivity. L‐lactate dehydrogenase was identified as the key enzyme forming valine's α‐hydroxy acid.

## Graphical Abstract and Lay Summary

Keto isoleucine and keto leucine are amino acid precursors, which can be used by CHO cells to produce biotherapeutics. The authors demonstrate that the supplementation of the keto acid derivatives in cell culture feeds alters the levels of related by‐products formed during the process. Overall, the findings highlight that despite by‐product formation, keto isoleucine and keto leucine can have a positive impact on the production of biotherapeutics.

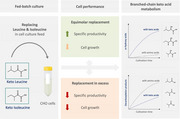

**Abbreviations**
AA: amino acid
ACN: acetonitrile
ACOT: acyl‐CoA thioesterase
AEX: anion exchange chromatography
BCAA: branched‐chain amino acid
BCAT: branched‐chain aminotransferase
BCHA: branched‐chain α‐hydroxy acid
BCKA: branched‐chain α‐keto acid
BCKDH: branched‐chain keto acid dehydrogenase
CCF: cell culture feed
CCM: cell culture medium
CHO: Chinese hamster ovary
CV: column volumes
DATAN: diacetyl‐L‐tartaric anhydride
dcBCKA: products of branched‐chain keto acid decarboxylation
dcKI: product of α‐keto isoleucine decarboxylation/2‐methylbutyric acid
dcKL: product of α‐keto leucine decarboxylation/3‐methylbutyric acid/Isovaleric acid
dcKV: product of α‐keto valine decarboxylation/2‐methylpropionic acid/Isobutyric acid
EDC: N‐(3‐dimethylaminopropyl)‐N′‐ethylcarbodiimide hydrochloride
ESI: electrospray ionization
Glu: glutamate
GS: glutamine synthetase
HA: α‐hydroxy acid
HI: α‐hydroxy acid of isoleucine/2‐hydroxy‐3‐methylpentanoic acid
HL: α‐hydroxy acid of leucine/2‐hydroxy‐4‐methylpentanoic acid/2‐hydroxyisocaproic acid
HV: α‐hydroxy acid of valine/2‐hydroxy‐3‐methylbutyric acid/2‐hydroxyisovaleric acid
Ile: isoleucine
ISTD: isotopically labeled standard
IVC: integral of viable cells
KA: α‐keto acid
KG: ketoglutarate
KI: α‐keto isoleucine/2‐keto‐3‐methylpentanoic acid
KL: α‐keto leucine/2‐keto‐4‐methylpentanoic acid
KV: α‐keto valine/2‐keto‐3‐methylbutyric acid
LC: liquid chromatography
LDH: lactate dehydrogenase
Leu: leucine
mAb: monoclonal antibody
MeOH: methanol
MS: mass spectrometry
MS/MS: tandem mass spectrometry
MTBE: methyl tert‐butyl ether
NAD(H): (reduced) nicotinamide adenine dinucleotide
NADP(H): (reduced) nicotinamide adenine dinucleotide phosphate
O‐BHA: O‐benzylhydroxylamine
ROS: reactive oxygen species
SEC: size exclusion chromatography
tris‐HCl: tris(hydroxymethyl)aminomethane hydrochloride
U(H)PLC: ultra (high) performance liquid chromatography
UV: ultraviolet
Val: valine
VCD: viable cell density

## 1 Introduction

In 2023, the United States Food and Drug Administration [1] approved 12 therapeutic antibodies produced in bioprocesses using Chinese Hamster Ovary (CHO) cells, highlighting the importance of this cell expression system for the pharmaceutical industry. The increasing demand for biotherapeutics targeting diseases, such as cancer, requires constant development and improvement of culture processes to enhance CHO productivity while lowering production costs and ecological footprint. Cell culture media (CCM) and feeds (CCF) are key factors in the development of next generation processes, as they contribute to performance parameters, such as cell growth, viability, and productivity, as well as critical quality attributes, such as glycosylation, aggregation, and charge variants [2, 3, 4]. The concentration of media formulations is an essential strategy to intensify processes and contribute to these efforts. Even though cystine and tyrosine are the first critical media components in limiting further CCM concentration due to their low solubility, the branched‐chain amino acids (BCAA) isoleucine (Ile) and leucine (Leu) were identified as the next critical molecules [5].

For CHO cultivation, the N‐lactoyl and α‐keto acid (BCKA) derivatives of Ile and Leu were described as bioavailable precursors for their amino acids (AA), and their replacement allowed for further concentration of CCFs [6]. For BCKAs, the amination reaction to Ile and Leu is known to be catalyzed by the branched‐chain aminotransferase (BCAT, EC 2.6.1.42) [7]. While glutamate (Glu) is typically considered the amino group donor for BCKA amination, resulting in the formation of α‐ketoglutarate (KG), investigations in CHO lysates highlighted that the respective other BCAAs (e.g., valine (Val)) are preferred substrates to provide the amino group. The understanding of this interdependancy between BCAAs and BCKAs is essential to optimize their concentrations in CCM and CCF and develop a suitable formulation. In previous experiments, the replacement of Ile and Leu by their α‐keto acids, keto isoleucine (KI) and keto leucine (KL), when used above a defined concentration, induced a decreased growth and productivity in different CHO cell lines, but the underlying mechanisms for the observed effects remain unclear [5].

Recently, various metabolites derived from BCAAs were investigated in the context of growth inhibition and impact on specific productivity during CHO cultivation. Isobutyric (dcKV), isovaleric (dcKL), and 2‐methylbutyric acid (dcKI) are small organic acids, which are the hydrolyzed products of acyl‐CoA species formed from BCKA decarboxylation by branched‐chain keto acid dehydrogenase (BCKDH) complex [7]. Mulukutla et al. demonstrated that all three individual decarboxylation products of BCKAs have substantial inhibitory effects on cell growth at concentrations above 1 mM, which can be typically found in spent media of CHO cultivations [8, 9, 10]. Harrington et al. further highlighted that decreased cell growth upon addition of dcKL and dcKV to CHO cultures was concurrent with an increase in specific productivity [11].

Similarly, the α‐hydroxy acids (BCHA) of leucine and valine, namely 2‐hydroxyisocaproic (HL) and 2‐hydroxyisovaleric acid (HV), were described as BCAA metabolites in CHO cells and were suggested to be growth‐inhibiting by‐products [8, 12, 13]. Recently, a study by Gonzalez et al. using ^13^C‐labeled amino acids confirmed the additional formation of the α‐hydroxy acid of isoleucine (HI) in CHO cells. L‐lactate dehydrogenase (L‐LDH) was suggested as a potential enzyme catalyzing the reduction of all BCKAs forming BCHAs [14]. Notably, in this study, only high concentrations (5 mM) of spiked HI showed growth‐inhibiting effects, while no change was observed compared to an unspiked control for the same concentrations of HL and HV. Therefore, the impact of BCHAs on cell growth remains controversial.

In this work, the formation of BCHAs and their impact on culture performance were investigated upon replacement of Ile and Leu by their respective keto acids (KAs). Therefore, fed‐batch experiments were performed, and BCAAs, BCKAs, and BCHAs were quantified using dedicated liquid chromatography–based methods. The ratio of BCHA stereoisomers was studied, and spiking experiments with the respective BCHA stereoisomers were performed to assess their impact on cell growth and titer. In an extensive investigation of BCHA formation in CHO cells, the capability of isolated L‐LDH to form BCHAs from BCKAs, utilizing NADH as an electron donor, was studied. Oxamic acid, an L‐LDH inhibitor, was also spiked to the basal medium during a fed‐batch to study the involvement of endogenous L‐LDH in BCHAs formation. Lastly, a two‐dimensional chromatographic fractionation of CHO lysates followed by a catalytic assay was performed to link specific enzymes present in isolated fractions to the BCHA formation. Altogether, we showed that BCKA supplementation leads to substantially increased BCHA formation, discovered opposing enantiomer ratios formed in the case of HV and HL and identified L‐LDH as the enzyme being responsible for the formation of BCHA S‐enantiomers, especially HV.

## 2 Materials and Methods

### 2.1 Chemicals, Reagents, and Cell Lines

The following reagents and standard compounds were obtained from Merck KGaA (Darmstadt, Germany): sodium chloride, acetic acid, tris(hydroxymethyl)aminomethane hydrochloride (tris‐HCl), ammonium formate, formic acid, L‐lactate dehydrogenase (LDH) from bovine heart, PBS, CytoBuster, NADH, NADPH, acetonitrile (ACN), methanol (MeOH), methyl tert‐butyl ether (MTBE), iodoacetamide, norvaline, O‐benzylhydroxylamine (O‐BHA), N‐(3‐dimethylaminopropyl)‐N′‐ethylcarbodiimide hydrochloride (EDC), (+)‐O,O′‐diacetyl‐L‐tartaric anhydride (DATAN), 2‐methylpropionic acid, 2‐methylbutyric acid, 3‐methylbutyric acid, 3‐methyl‐2‐oxopentanoic acid sodium salt, 4‐methyl‐2‐oxopentanoic acid sodium salt, 3‐methyl‐2‐oxobutyric acid sodium salt, ^13^C_5_‐3‐methyl‐2‐oxobutyric acid, ^13^C_6_,^15^N‐L‐leucine, 2‐hydroxy‐3‐methylbutyric acid, 2‐hydroxy‐3‐methylpentanoic acid, and 2‐hydroxy‐4‐methylpentanoic acid. ^13^C_6_‐4‐methyl‐2‐oxopentanoic acid sodium salt was sourced from Cambridge Isotope Laboratories (Tewksbury, MA, USA). d_7_‐2‐methylpropionic acid, d_3_‐2‐methylbutyric acid, d_7_‐2‐hydroxy‐3‐methylbutyric acid, d_9_‐3‐methylbutyric acid, d_8_‐3‐methyl‐2‐oxopentanoic acid, d_3_‐4‐methyl‐2‐oxopentanoic acid, and d_3_‐L‐2‐hydroxy‐4‐methylpentanoic acid were obtained from Toronto Research Chemicals (Toronto, ON, Canada). d_10_‐2‐hydroxy‐3‐methylpentanoic acid (mixture of all four diastereomers) was synthesized internally from d_10_‐isoleucine sourced from CDN Isotopes (Teddington, UK). (−)‐Diacetyl‐D‐tartaric anhydride was purchased from Santa Cruz Biotechnology (Dallas, TX, USA). Sodium oxamate was ordered from Cayman Chemicals (Ann Arbor, MI, USA).

Chemically defined Cellvento 4CHO medium and ModiFeed Prime COMP (both commercially available from Merck Life Science KGaA, Darmstadt, Germany) were used in this study to produce a monoclonal antibody (mAb) with the aid of a glutamine synthetase (GS) expressing CHOK1 cell line. Ile and Leu were purchased from Merck Life Science KGaA (Darmstadt, Germany), and the sodium salts of KI and KL for cell culture were sourced in‐house.

### 2.2 Fed‐Batch Culture

CHOK1 cells, transfected with a plasmid containing the GS gene, were cultivated in Cellvento 4CHO medium without supplementation of L‐glutamine. Cellvento ModiFeed Prime COMP was depleted in Ile and Leu to allow individual supplementation or exchanges with KL and KI. Fed‐batch processes were performed in 50‐mL spin tubes with a vented cap (TPP, Trasadingen, Switzerland) at 37°C, 5% CO_2_, 80% humidity and a rotation speed of 320 rpm. For feeding, 2.75%, 4.1%, 6%, 6%, and 2.75% (v/v) of the total volume were added on days 3, 5, 7, 10, and 13, respectively. Glucose concentrations were maintained by the addition of specific volumes of a 400‐g/L glucose stock solution on demand up to 6 g/L during the week and up to 14 g/L before the weekend. Cell counts and viability were measured with a Vi‐CELL XR 2.04 cell counter (Beckman Coulter, Fullerton, CA, USA). The seeding density was 2 × 10^5^ cells/mL in 30 mL starting volume. Experimental conditions were performed with four replicates, if not stated otherwise. Spent media analysis, including glucose, IgG, ammonia, iron, lactate, and pyruvate, was performed with the bioprocess analyzer Cedex Bio HT (Roche, Mannheim, Germany) after centrifugation of the sample for 2.5 min with 2,000 rcf.

### 2.3 Quenching and Extraction for Analysis of Intracellular Metabolites

For the harvest of cells for intracellular analyses, the suspension containing 10^7^ cells was quenched by gentle mixing with four times the suspension's volume of Normal Saline (pre‐chilled at 0.5°C), rapid centrifugation with 1000 rcf at 4°C for 1 min, immediate removal of supernatant and subsequent snap‐freezing in liquid nitrogen.

For metabolite extraction, the quenched pellets were resuspended with 2 mL ACN–MeOH–water (2:2:1 v/v/v). 200 µL of the suspension was centrifuged at 10,000 rcf at 4°C for 5 min, and the supernatant was removed. For normalization, the obtained pellet was used for protein quantification using the Pierce Detergent Compatible Bradford Assay Kit by Thermo Fisher Scientific (San Jose, CA, USA) following the metabolite extraction. 600 µL aliquots of the suspension were transferred to clean tubes and shaken with 1600 rpm at 4°C for 15 min, before the cell debris were pelleted by centrifugation with 18,213 rcf at 4°C for 15 min. Subsequently, 480 µL of the supernatant were transferred to a clean tube and dried using a vacuum centrifuge. The extracts were resolubilized in 60 µL water by shaking with 1,600 rpm at 4°C for 15 min. Upon centrifugation for the removal of potential insoluble components, the extracts were subjected to analyte quantification, as elaborated below.

### 2.4 Quantification of AA

For amino acid quantification, spent media samples and resolubilized intracellular metabolite extracts were prepared and analyzed using the AccQ‐Tag method. Briefly, spent media and metabolite extracts were diluted in 0.1 M hydrochloric acid by a factor of 10 and 2, respectively, and supplemented with 20 mM iodoacetamide and 250 µM norvaline, before incubation for 45 min at room temperature. Subsequently, alkylated samples were derivatized using the AccQ‐Tag derivatization kit by Waters (Milford, MA, USA) according to the manufacturers protocol and quantified by UPLC–UV analysis.

### 2.5 LC–MS Quantitation of BCHA

#### 2.5.1 Sample Preparation

For BCHA quantification, 10 µL of spent medium were mixed with 240 µL of ice‐cold ACN–water (1:3 v/v) with formic acid (0.1% v/v) followed by centrifugation at 18,213 rcf for 1 min. The spent media samples and the resolubilized metabolite extracts were further diluted with water to obtain analyte concentrations in the calibrated range and were supplemented with isotopically labeled standard (ISTD), resulting in final ISTD concentrations of 150 ng/mL. Calibrants containing all analytes ranging from 2.5 to 500 ng/mL were prepared with the same ISTD levels.

#### 2.5.2 LC–MS/MS Analysis

BCHAs were quantified in diluted samples using a UHPLC (1290 Infinity II, Agilent, Waldbronn, Germany) system coupled to a triple quadrupole mass spectrometer (G6495C, Agilent, Waldbronn, Germany) with electrospray ionization (ESI) in negative ionization mode (ESI‐). 2 µL of sample were subjected to chromatographic separation on an Acquity UPLC HSS T3 column (150 × 2.1 mm, 1.8 µm, Waters, Milford, MA, USA) with a two‐eluent gradient at a flow rate of 300 µL/min and a column temperature of 40°C. Eluent A consisted of 20 mM ammonium formate in water with 0.1% v/v formic acid, and eluent B was LC–MS grade methanol. The gradient was applied as follows (min/% B): 0/5, 2/5, 4/20, 6/30, 14/40, 14.5/100, 16.5/100, 16.6/5, 18/5. LC–MS parameters are summarized in Table S1. Data analysis was performed using MassHunter Software “Quantitative Analysis” by Agilent.

### 2.6 LC–MS Quantitation of BCKA and Decarboxylation Products

#### 2.6.1 O‐BHA Derivatization

The sample preparation for quantification of the three BCKAs, their respective decarboxylation products, and KG was modified from Lai et al. [8]. Briefly, spent media samples were diluted in ice‐cold ACN–water (1:3 v/v) with formic acid (0.1% v/v) by a factor of 50, 150, or 300 to obtain analyte concentrations in the calibrated range, followed by a centrifugation step at 18,213 rcf for 1 min. For the determination of intracellular metabolites, resolubilized extracts were diluted in water by a factor of 5. Calibrants were prepared in the range of 10–10,000 ng/mL (concentrations before derivatization), and a mix of stable isotope‐labeled analytes was prepared with each 3 or 1 µg/mL for the three decarboxylation products and the remaining ISTD compounds, respectively. For derivatization, 10 µL of supernatant or calibrants were mixed with 10 µL ISTD solution and 60 µL ACN, before each 10 µL of O‐BHA and EDC (both 0.1 M in MeOH) were added. The mixtures were incubated at RT for 1 h at 600 rpm. For analyte extraction, 1 mL MTBE and 150 µL purified water were added. The mixture was briefly vortexed and subsequently centrifuged for 1 min at 18,213 rcf. 200 µL of the organic phase were transferred to clean microcentrifuge tubes, dried with the aid of a vacuum centrifuge and ultimately reconstituted in 200 µL of MeOH–water (1:1 v/v) for LC–MS/MS analysis. Calibration standards and samples were diluted 1:100 through the described derivatization step, resulting in a final calibration range of 0.1–100 ng/mL.

#### 2.6.2 LC–MS/MS Analysis

Derivatized BCKAs and decarboxylation products were quantified by LC–MS/MS in positive ionization mode (ESI+) using the same instrumental setup by Agilent as for the BCHA quantitation. 5 µL were injected on a Phenomenex Luna Omega C18 column (100 × 2.1 mm, 1.6 µm, 100 Å) with 500 µL/min at 45°C and separated similarly to Lai et al. [8] using water with 0.1% formic acid (A) and methanol (B) with a gradient as follows (min/%B): 0/10, 0.5/10, 5/55, 7.5/65, 8/85, 9.5/85, 9.6/95, 11/95/ 11.3/10, 12.3/10. LC–MS parameters are summarized in Table S2. Data analysis was performed using MassHunter Software “Quantitative Analysis” by Agilent.

### 2.7 Determination of BCHA Stereoisomers by LC–MS

#### 2.7.1 DATAN Derivatization

For analysis of BCHA stereoisomers, the samples were derivatized with enantiomerically pure DATAN obtaining diastereomeric esters, which are separable by conventional RPLC methods. The method was derived from Pandey et al. [15]. After the addition of 40 µL ISTD solution containing 500 µM of stable isotope labeled HV (racemic) and HI (mixture of all four diastereomers), 40 µL spent medium were deproteinized by the addition of 120 µL ACN (pre‐chilled at −20°C) and subsequent centrifugation at 18,213 rcf and 4°C for 2.5 min. Two aliquots of 95 µL of the supernatant were dried by vacuum centrifugation. The dried residues were reconstituted in 100 µL (+)‐ or (−)‐DATAN reagent, which was prepared with 75 mg/mL in ACN–acetic acid (80:20, v/v), and subsequently incubated at 75°C with 1000 rpm for 2 h. The derivatized samples were centrifuged at 18,213 rcf for 2.5 min, and 95 µL of the obtained supernatant was transferred to a clean tube and dried by vacuum centrifugation. The residue was reconstituted in 100 µL water–ACN (80:20 v/v) by shaking for 5 min with 1,400 rpm and subsequently centrifuged for 2.5 min at 18,213 rcf, before the supernatant was analyzed by LC–MS.

#### 2.7.2 LC–HRMS Analysis

The reconstituted samples were separated using UHPLC (Vanquish, Thermo Fisher Scientific, San Jose, CA, USA) coupled to an ESI‐Q‐ToF mass spectrometer (Impact II, Bruker Daltonics, Bremen, Germany). 4 µL of sample were loaded in 100% buffer A (20 mM ammonium formate/0.1% formic acid) onto a ZORBAX RRHD Eclipse Plus C18 (1.8 µm, 100 × 2.1 mm, Agilent) with 600 µL/min at 40°C. A 28‐min linear gradient using acetonitrile as eluent B was applied as follows (min/%B): 0/0, 2/0, 20/20, 24/100, 25/100, 25.1/0, 28/0. The MS acquisition was conducted in positive mode, with a capillary voltage of 4500 V and an end plate offset of 500 V. The nebulizer and dry gas (250°C) were set at 1.4 bar and 9.0 L/min, respectively. MS spectra were obtained in the *m*/*z* range of 50–1000 at a scan rate of 2 Hz in MS mode for analyte quantification. Ratios of stereoisomers were determined by use of peak areas after normalization to the ISTDs (d_7_‐HV for HV, d_10_‐HI for HI and HL).

### 2.8 LDH Assay

To investigate the formation of BCHAs catalyzed by L‐LDH (P/N: L2625, Merck, Darmstadt, Germany), 400 µL of a PBS solution containing 8 mM NADH, 10 U/mL L‐LDH and each 2 mM of KI, KL, and KV were incubated for 3 h at 37°C. Furthermore, the same condition was performed (1) spiked with 20 mM sodium oxamate and (2) without L‐LDH as a negative control. After 3 h, 80 µL aliquots were taken and 20 µL of 1 M HCl was added to quench the reaction. Subsequently, the BCHA content was determined by LC–MS.

### 2.9 Lysate Assay

To investigate the formation of BCHAs in CHO lysates, CHOK1 GS cells were lysed with CytoBuster (100 µL per 2 × 10^7^ cells) and dialyzed with PBS using a Slide‐A‐Lyzer (3 kDa, 15 mL) cassette (Thermo Fisher Scientific, San Jose, CA, USA) to remove small molecules. 200 µL of a PBS solution containing 5 mg/mL lysate, each 2 mM of KI, KL, and KV, and 8 mM NADH or NADPH were incubated for 4 h at 37°C. After incubation, 50 µL of 1 M HCl were added to quench the reaction and to precipitate proteins. The suspension was centrifuged at 18,213 rcf for 2.5 min, before the supernatant was subjected to LC–MS for BCHA quantitation and determination of stereoisomer composition.

### 2.10 Two‐Dimensional Chromatographic Fractionation of CHOK1 GS Lysate

Using the ÄKTA avant chromatography system by Cytiva (Marlborough, MA, USA), CHOK1 GS lysate, prepared equivalent to the lysate assay, was fractionated using (1) size exclusion chromatography (SEC) and (2) anion exchange chromatography (AEX) to identify enzymes catalyzing the formation of HA from BCKA and NADH. Subsequently, the fractions’ ability to catalyze this reaction was evaluated by LC–MS and the proteins in the fractions were determined by means of a proteomic approach.

For the lysate fractionation by SEC, a Superdex 200 Increase 10/300 GL column (Cytiva, Marlborough, MA, USA) was operated isocratically with PBS at a flowrate of 0.75 mL/min. 1 mL of lysate, corresponding to 11 mg protein, was injected onto the equilibrated column in duplicate. 1‐mL fractions were collected for approximately 1.2 column volumes (CV), while the UV signal was monitored at 280 nm. Fractions were supplemented with Halt Protease and Phosphatase Inhibitor Cocktail (Thermo Fisher Scientific, San Jose, CA, USA) to prevent protein degradation.

A selection of fractions collected from the SEC run was further processed by means of AEX chromatography. Prior to the separation, the matching fractions of the two SEC runs were pooled, and the buffer of the fractions was partially exchanged with water, in order to reduce the salt content prior to AEX. Approximately, 1 mL of individual fractions was separated on a Mono Q 5/50 GL (Cytiva, Marlborough, MA, USA) applying a salt gradient using 20 mM tris‐HCl at pH 7 without (buffer A) and with (buffer B) 1 M sodium chloride. The flowrate was set to 2 mL/min, and following a column wash of 2 CV after injection, a linear gradient from 0% to 100% buffer B was applied over 20 CV, which was held at 100% B for 3 CV. Following elution, the column was washed with 5 CV buffer A. 1‐mL fractions were collected from the start of the sample application until the start of the wash step and the separation was monitored permanently with the UV detector set to 280 nm. Fractions were supplemented with Halt Protease and Phosphatase Inhibitor Cocktail to prevent protein degradation.

To determine the fraction's capability of catalyzing the formation of BCHA from BCKA and NADH, 230 µL of the obtained fractions were mixed with 10 µL of 100‐mM NADH and 10 µL of a solution containing each 50 mM KI, KL, and KV. This mixture was then incubated for 24 h at 37°C. Then, 250 µL of ACN (pre‐chilled at ‐20°C) were added and the samples were centrifuged at 5,000 rcf for 5 min. The supernatants were diluted in water by a factor of 5, before relative quantitation of BCHAs was performed using an LC–MS method, that was previously described for the quantitation of the respective BCKAs [5].

For relative protein quantitation, SEC fractions were diluted in water by a factor of 10, while AEX fractions were digested without prior dilution. Details of the tryptic digest and the consequent peptide quantitation by LC–MS are described in Supp. Methods 1. Label‐free MS quantification was performed using Progenesis QI for Proteomics by Waters (Milford, MA, USA). “Relative quantitation using non‐conflicting peptides” was used for quantitation and, ultimately, the quantified proteins and peptides were separately loaded to Tableau Data Analysis software (Tableau Software LLC, Seattle, WA, USA) for visualization. The abundance was plotted over the individual fractions, and heatmaps with the relative abundances per protein were created to highlight the fractions that showed the highest abundances for the individual proteins. These profiles were then visually compared to the fractions’ capability of reducing KV to HV in the presence of NADH as determined by the catalytic assay.

### 2.11 Statistical Analysis

Fed‐batch parameters are expressed as mean ± standard error of mean of four biological replicates unless stated otherwise. Quantitative data of metabolites determined by UPLC–UV or LC–MS are shown as mean ± standard error of two biological replicates unless stated otherwise. To compare cell culture parameters for fed‐batch experiments, the integral of viable cells (IVC), product titer, or metabolite concentrations until day 17 were determined by the GraphPad Prism 7 Software (GraphPad Software Inc., San Diego, CA, USA) for each biological replicate.

## 3 Results

### 3.1 High Concentrations of BCKAs Reduce Cell Growth and Titer

The impact of Leu, Ile, and their respective KA sodium salts, KI and KL, in modified Cellvento ModiFeed Prime was tested in fed‐batch using a CHOK1 GS cell line. The original feed formulation was depleted in Ile and Leu and subsequently supplemented with different amounts and combinations of Leu, Ile, KL, and racemic KI. The depleted feed served as a negative control to monitor Ile and Leu consumption from the medium, while feed supplemented with Ile and Leu (“Ile/Leu”) was considered the positive control. Five additional feeds were prepared as follows: the first two feeds contained Ile and KL (“Ile/KL”) or KI combined with Leu (“KI/Leu”), where the AA were replaced by their KAs in equimolar concentrations. In the remaining three feeds, Ile and Leu were replaced by both KI and KL in equimolar (“KI/KL”) as well as 1.5‐ or 2‐fold concentrations (“1.5KI/1.5KL,” “2KI/2KL”).

**FIGURE 1:**
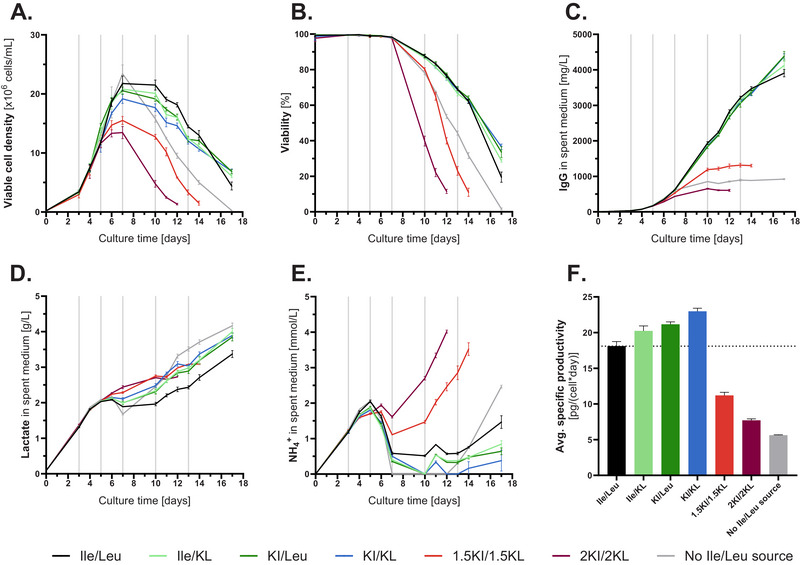
Viable cell density, viability, titer, concentrations of lactate and ammonia as well as average specific productivity obtained in fed‐batch with different combinations of keto and amino acids (*n* = 4). The positive control feed contained Ile and Leu, the negative control feed was depleted in Ile and Leu, while the other feeds were prepared by replacing Ile and Leu individually or in combination by their respective keto acids, either in equimolar, 1.5‐ or 2‐fold concentrations. In the basal medium, Ile and Leu were supplemented for all conditions. CHOK1 GS cells were cultivated in Cellvento 4CHO medium and 2.75%, 4.1%, 6%, 6%, and 2.75% (v/v) of modified Cellvento ModiFeed Prime were added on days 3, 5, 7, 10, and 13, respectively. (A) Viable cell density in 10^6^ cells/mL. (B) Viability in percent measured using ViCell XR. (C) IgG concentration in mg/L. (D) Lactate in g/L. (E) NH_4_^+^ in mmol/L measured using the Cedex Bio HT. (F) Average specific productivity in pg/(cell*day) determined by dividing the titer at the end of cultivation by the integral of viable cells (IVC). Grey vertical lines indicate feed supplementation.

Results (Figure 1) indicate that the replacement of Ile and Leu by their KA led to decreased peak viable cell densities (VCD) in a KA concentration‐dependent manner, for example, −13%, −29%, and −39% for KI/KL, 1.5KI/1.5KL, and 2KI/2KL, respectively. The viability of the negative control as well as 1.5KI/1.5KL and 2KI/2KL conditions decreased faster compared to the positive control and conditions with equimolar replacements of both or individual AAs. 1.5KI/1.5KL and 2KI/2KL conditions dropped below a viability of 20% on day 14 or 12, respectively, while the other conditions did not decline below this limit by day 17. Cytotoxicity assays demonstrated, that both KI and KL can induce toxicity (1) individually and (2) with comparable potency, as indicated by their LD_50_ values of 17.9 and 14.6 mM for KI and KL, respectively [data not shown]. While slight increases in titers were observed at the end of cultivation for conditions with equimolar replacements compared to the positive control, titers decreased harshly by −67% and −84% for the 1.5KI/1.5KL and 2KI/2KL conditions, respectively. Analysis of released N‐glycans and aggregation was performed for conditions with equimolar replacement of BCAAs by their BCKAs. While results indicated a slight increase in galactosylation upon BCKA supplementation, no impact on mAb aggregation was observed (Figure S1). During the growth phase, the net lactate production was higher with increased KA supplementation. The pattern observed with ammonia was different. While the equimolarly replaced conditions had lower ammonia levels compared to the positive control on day 7, the 1.5KI/1.5KL and 2KI/2KL conditions had higher concentrations, before they started to increase steeply as of day 10. Ultimately, these effects led to increasing average specific productivities for equimolarly replaced conditions, but in the case of 1.5KI/1.5KL and 2KI/2KL conditions, decreases of −38% and −57% were observed. Overall, these two conditions were quite distinct from the conditions with equimolar replacement with respect to the severity of impact on cell performance, indicating that high concentrations of BCKAs reduce CHO cell growth and titer. To assess the role of metabolic and oxidative stress in this performance reduction, intracellular NAD^+^/NADH ratios and reactive oxygen species (ROS) levels were determined (Figure S2). Ultimately, results indicated no substantial impact of the BCKA supplementation on NAD^+^/NADH ratios and ROS levels.

### 3.2 Available BCAAs Serve as Amino Group Donors for Supplemented BCKAs

To understand the mechanism of BCAA formation from BCKAs, the amination process was studied during fed‐batch cultivation. More specifically, analyses were performed (1) to investigate the intracellular availability of BCAAs upon BCKA supplementation and (2) to identify the main amino group donor(s). Therefore, the intracellular CHO content was extracted at multiple time points during the fed‐batch and analyzed using targeted LC–MS/MS for the quantification of BCKAs and KG. Additionally, the AAs were quantified using LC‐UV following AccQ‐Tag derivatization. These methods ensured sufficient chromatographic separation of structural isomers, yet the two enantiomers of KI were not separated and were therefore stated as a sum parameter.

**FIGURE 2:**
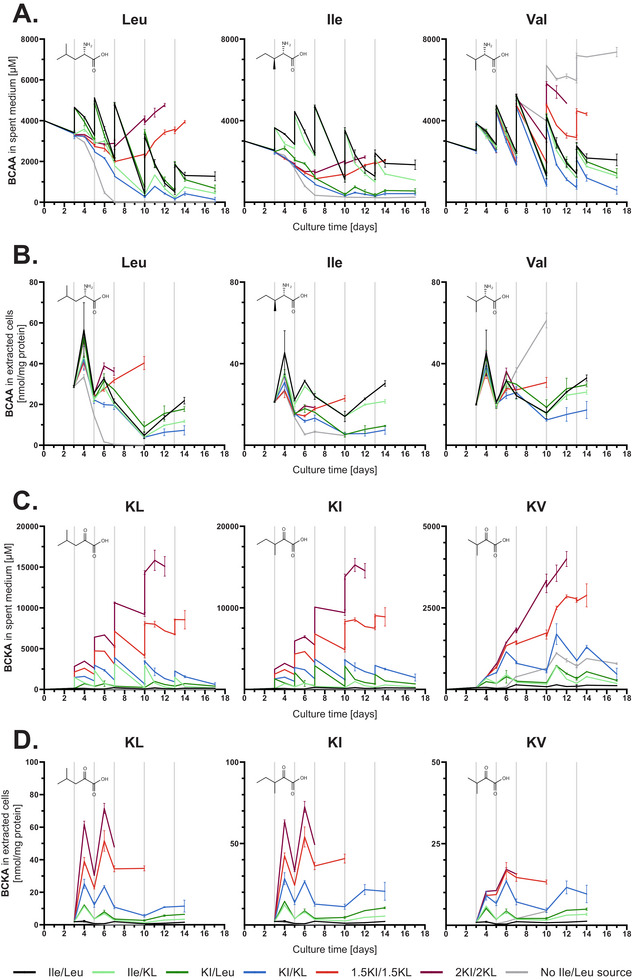
Extra‐ and intracellular levels of the three BCAAs and their BCKAs obtained in fed‐batch with different combinations of keto and amino acids (*n* = 2). BCAA and BCKA concentrations were determined by UPLC–UV following AccQ‐Tag derivatization and LC–MS/MS following O‐BHA derivatization, respectively. (A) Extracellular BCAA levels in µM. (B) Amount of intracellular BCAAs normalized to the total protein content in nmol/mg protein. (C) Extracellular BCKA levels in µM. (D) Amount of intracellular BCKAs normalized to the total protein content in nmol/mg protein. Grey vertical lines indicate feed supplementation.

None of the BCAAs were depleted extra‐ or intracellularly, as long as sources for Ile and Leu, namely either the BCAA or BCKA, were supplemented (Figure 2A,B). Hence, only the negative control supplemented with feed lacking Ile and Leu was fully depleted in Leu and showed the lowest amount of remaining Ile as of day 7 of cultivation. The KI/KL condition showed the lowest BCAA levels among the other conditions throughout the fed‐batch and, importantly, also the intra‐ and extracellular Val concentrations were decreased. 1.5KI/1.5KL and 2KI/2KL supplementation did not lead to a decrease in BCAAs. These two conditions had higher BCAA availability than the KI/KL condition, which was observed along with a general decrease in extracellular consumption of other essential AAs (Figure S3). Compared to KI/KL, the use of individual BCKAs showed slightly enhanced concentrations of the replaced BCAA, while the respective other BCAA, which was not replaced, was slightly decreased compared to the positive control. By the end of cultivation, Val levels were moderately decreased compared to the positive control, to a similar extent between Ile/KL and KI/Leu. Overall, the BCAA profiles indicate that (1) the decrease in cell growth and titer upon high concentrations of BCKAs is not linked to a reduced amination to BCAAs and (2) the BCAAs themselves are likely to serve as amino group donors for the supplemented BCKAs.

As a consequence of supplementing racemic KI, the non‐proteogenic diastereomer of Ile, namely Allo‐Ile, which substantially accumulated intra‐ and extracellularly in KI‐supplemented conditions (Figure S4), was studied in more detail. In the spent medium, the maximal concentration of 2.1 mM Allo‐Ile was detected in the KI/Leu condition at the end of culture on day 17. Concurrently, a lower concentration of extra‐ and intracellular Ile was observed, when it was replaced by KI, compared to conditions supplemented with Ile. As an example on day 10, the lowest concentration of Ile in the positive control and the KI/Leu condition was 1.1 and 0.4 mM, respectively. Overall, these findings suggest that a substantial portion of supplemented KI was aminated to form Allo‐Ile leading to lower Ile availability.

Quantification of BCKAs indicated that their levels were typically very low intra‐ and extracellularly when the cells were supplemented with BCAAs only (Figure 2C,D). The extracellular concentrations for KL, KI, and KV were typically below 0.3 mM, while intracellularly, the content was below 3 nmol/mg protein in the positive control. Replacement of Ile and Leu by their BCKAs led to a substantial increase in KI and KL, but also KV intra‐ and extracellularly. While equimolar replacement showed a balanced pattern of supplementation and consumption with the upper limit of 4 mM KI or KL in the spent medium, the use of 1.5KI/1.5KL and 2KI/2KL led to an accumulation of KI and KL. In the 2KI/2KL condition, extracellular levels reached 10 mM after feeding on day 10, before the viability started to drop rapidly. These results highlight that the exogenous BCKA supplementation substantially exceeded the concentrations that CHO cells are typically exposed to when only BCAAs are supplemented.

Interestingly, in conditions with individual replacement of one BCAA by its KA, the respective other BCKA, which was not supplemented, was also substantially increased both intra‐ and extracellularly. For example, concentration spikes for KI were observed intracellularly after KL addition (Figure 2D, light green), leading to almost identical KI levels as for the KI supplemented conditions (Figure 2D, dark green). Similarly, KV was rapidly increased intracellularly upon KI and KL supplementation in a dose‐dependent manner and accumulated up to 2 mM in the spent medium. In 1.5KI/1.5KL and 2KI/2KL conditions, net accumulations of KV were observed in the supernatant, reaching approximately 3 and 4 mM, respectively, before the conditions were discarded due to low viability. Considering the interdependency between the three BCAAs and BCKAs, any of the available BCAAs may be utilized to aminate the supplemented BCKAs. Specifically, Val may be favored when both Ile and Leu are replaced simultaneously and thus adjustment of the Val concentration in the formulation might be required.

Lastly, to conclude the investigations on the amination process, we quantified intra‐ and extracellular levels of Glu and KG, the well‐known co‐substrate and product of BCAA/BCKA transamination by BCAT. Compared to the positive control, replacement of Ile and Leu by their BCKAs led to increased intracellular levels of Glu and KG (Figure S5). 1.5KI/1.5KL and 2KI/2KL supplementation led to increased extracellular concentrations of both Glu and KG. While equimolar replacement of Ile and Leu, both individually and simultaneously, also resulted in an increase in extracellular KG, extracellular Glu was slightly reduced compared to the positive control. Hence, based on these results, a contribution of Glu to the amination of KI and KL cannot be excluded.

### 3.3 Increased Accumulation of BCHAs, but Not of BCKA Decarboxylation Products

As the α‐hydroxy acid derivatives of Val (HV) and Leu (HL) as well as the decarboxylation products of KI (dcKI), KL (dcKL), and KV (dcKV) were recently associated with decreased cell growth [9, 10, 11, 12, 13], targeted LC–MS/MS methods were established to quantify all three BCHAs and dcBCKAs extra‐ and intracellularly. The dcBCKAs were quantified after O‐BHA derivatization. To avoid false‐positive results and ensure sufficient chromatographic resolution to discriminate structural isomers, the BCHAs were analyzed with a dedicated method, as HI and HL were not sufficiently separated by use of the O‐BHA derivatization method. Eventually, BCHA quantitation did not use pre‐column derivatization and was performed with a different separation method. In general, stereoisomers were not distinguished by these two methods, hence, the obtained quantitative results were sum parameters of the respective stereoisomers, that is, each two enantiomers of HL, HV, dcKI, as well as the four diastereomers of HI. In order to quantify ratios of BCHA stereoisomers, a third method using DATAN derivatization was developed.

**FIGURE 3:**
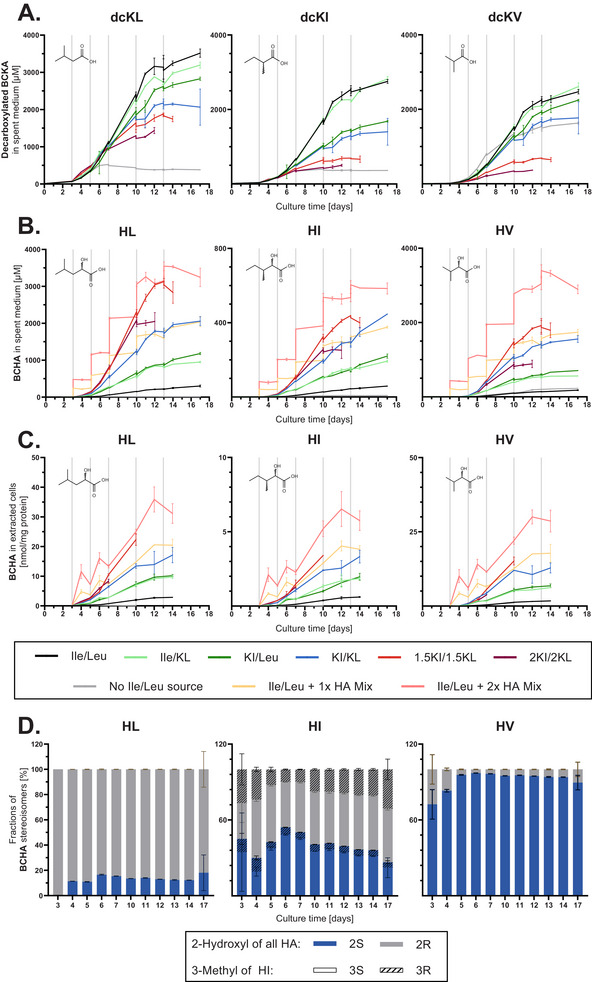
Quantification of BCKA‐derived metabolites in fed‐batch using two targeted LC–MS/MS approaches. The BCHA measurements included the quantification of two additional conditions, where R‐HL, S‐HV, and a mixture of HI diastereomers were spiked to the feed containing Ile and Leu at two concentration levels. (A) Concentrations of the BCKAs’ decarboxylation products in the spent medium in µM (*n* = 2). (B) Concentrations of BCHAs in the spent medium in µM (*n* = 2). (C) Intracellular amounts of BCHAs normalized to the total protein content in nmol/mg protein (*n* = 2). Grey vertical lines in (A)–(C) indicate feed supplementation. (D) Fractions of the (extracellular) BCHAs’ stereoisomers in the condition supplemented with equimolar concentrations of KI and ^13^C_6_‐KL (*n* = 4) in the feed and replacement of ^15^N,^13^C_6_‐Leu in the basal medium.

The intra‐ and extracellular quantification of the BCKAs’ decarboxylation products indicated decreasing formation with increasing amount of supplemented BCKAs (Figure 3A /Figure S6). By the end of cultivation on day 17, in the positive control, dcKL, dcKI, and dcKV accumulated extracellularly up to 3.5, 2.8, and 2.5 mM, respectively. For the KI/KL condition, the maximum concentrations were 2.1, 1.4, and 1.8 mM for dcKL, dcKI, and dcKV, respectively, while the concentrations in the 2KI/2KL condition reached only 1.4, 0.50, and 0.37 mM by day 12, before the condition was discarded due to low viability. To decouple the effects of decreased dcBCKA synthesis from the reduction in VCD, the average specific formation rates for these decarboxylated products were calculated based on their final concentrations and the IVC (Figure S7). Results indicate that the quantity of dcKI and dcKV formed per cell per day decreased upon supplementation of KI and KL, in particular in case of 1.5KI/1.5KL and 2KI/2KL conditions (Figure S7). In the case of dcKL, the specific production rate was similar to the positive control in these two conditions. These results indicate that both the absolute concentration and the cell‐specific formation rate of dcBCKAs decreased despite the elevated availability of BCKAs, suggesting that the decrease in cell growth and titer upon high concentrations of BCKAs (Figure 1) is not linked to the formation of BCKA decarboxylation products.

In contrast to decreased dcBCKA levels, a substantial increase in all three BCHAs was observed upon BCKA supplementation, despite the decreased VCDs (Figure 3B,C). The intra‐ and extracellular BCHA levels showed steady accumulation during the cultivation and, overall, the intra‐ and extracellular data presented the same trends. After 17 days, the positive control supplied with Ile and Leu reached 0.30, 0.18, and 0.06 mM of HL, HV, and HI in the spent medium, while KI/KL supplementation resulted in 2.1, 1.6, and 0.45 mM, respectively. The produced BCHAs represented 19%, 14%, and 5% of the amount of KL, Val, and KI supplemented in the feed. While all three BCHAs showed substantially increased accumulation upon BCKA supplementation, HV and HL appeared to be by far more abundant than HI. Accordingly, a more substantial portion of Val and Leu, or their KAs, was directed towards the formation of these metabolic products, which might impact overall cell growth and productivity.

To explore the mechanism of BCHA formation and give hints regarding the involved enzymatical reaction(s), the ratio of predominant BCHA stereoisomers was quantified using a dedicated LC–MS method. The basis of this approach was the derivatization of the hydroxy groups with defined enantiomers, namely (+)‐ or (−)‐DATAN, forming diastereomeric esters (Figure S8), a requirement for the potential separation with achiral chromatography columns. The protocol for the derivatization and chromatographic method was modified from Pandey et al. [15]. Despite the separation of HL and HV enantiomer pairs, separation with reversed‐phase chromatography did not allow for the separation of all four HI diastereomers, as two species co‐eluted. Furthermore, the peaks of structural isomers HI and HL overlapped (Figure S9). Therefore, another KI/KL condition was included in the fed‐batch experiment, where both the Leu in the basal medium and the KL in the feed were replaced by ^15^N,^13^C_6_‐Leu, and ^13^C_6_‐KL, respectively. This use of labeled HL precursors enabled the discrimination of HI and HL through MS detection and confirmed the presence of six ^13^C‐atoms in the structure of HL aligning with findings from Gonzalez et al. [14]. Additionally, similar experiments conducted with labeled KV and KI confirmed the presence of five and six ^13^C‐atoms in HV and HI, respectively [data not shown]. Finally, the derivatization was performed with both DATAN enantiomers in parallel, which resulted in the interexchange of the peaks of HI enantiomers, in order to resolve and calculate the fractions of all four HI diastereomers. As an example, the (2S,3S) and (2R,3R)‐stereoisomers changed positions when derivatized with either enantiomer of DATAN (Figure S10).

The determination of stereoisomer fractions of the individual BCHAs indicated that different species were favored in the reduction of the three KAs in culture using CHOK1 GS (Figure 3D). While the D‐enantiomer (2R‐conformation) was the predominant species for HL with a fraction slightly below 90%, the L‐enantiomer (2S‐conformation) was favored in the case of HV with a fraction above 90%. With its two chiral centers, the stereoisomer composition of HI was more complex by nature. The majority of HI (70%–80%) was comprised of the 3S‐diastereomers (analogous to Ile, not Allo‐Ile) with a fairly equal ratio of the 2S‐ and 3S‐conformation, that varied slightly over time. Overall, these results indicate that the stereoisomer composition varies substantially between the three BCHAs and that their formation is likely catalyzed by different enzymes.

### 3.4 Impact of BCHA on Cell Growth and Specific Productivity

To investigate the impact of the BCHAs on cell growth, viability, and IgG titer, a fed‐batch was performed and a combination of D‐HL, L‐HV and a mixture of all HI diastereomers was spiked to ModiFeed Prime containing Ile and Leu. To mimic the BCHA concentration profile obtained in the KI/KL condition, the spiked concentrations corresponded to the fractions of supplemented KI, KL, and Val, which were ultimately converted into the respective BCHAs. This condition was referred to as “Ile/Leu 1x HA Mix.” Furthermore, a condition with twice these BCHA concentrations, representing a worst‐case scenario, was introduced (“Ile/Leu 2x HA Mix”), and the extra‐ and intracellular levels of the BCHAs were quantified.

**FIGURE 4:**
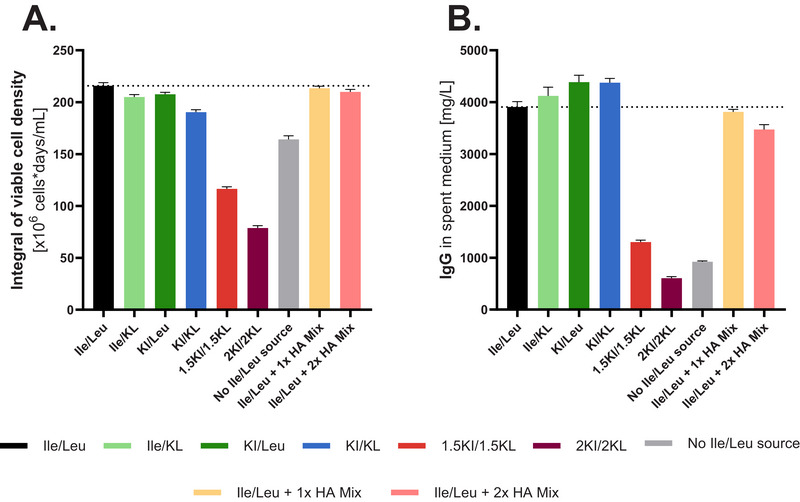
Impact of BCHA spiking at representative concentrations on the integral of viable cells (IVC) and titer. (A) IVC until the end of culture based on viable cell densities measured using the ViCell XR (*n* = 4). (B) Total IgG concentration in mg/L measured using the Cedex Bio HT (*n* = 4).

Extra‐ and intracellularly, the BCHA quantification indicated similar concentration profiles of all three species for the 1x HA Mix and the KI/KL condition (Figure 3B,C). Due to the stepwise supplementation of the BCHAs through the feed, the spiked condition tended to have higher concentrations than the referenced condition. The two‐fold amount of spiked BCHAs showed the overall highest levels extra‐ and intracellularly. In terms of cell culture performance, IVC did not change noticeably (Figure 4A) upon BCHA spiking, nor did the viability [data not shown]. IgG titers (Figure 4B) only slightly decreased upon spiking with the two‐fold concentrated BCHA mix, hence the average specific productivity was slightly lower. Overall, this experiment indicates a low impact of BCHAs on cell performance despite the substantial accumulation of these metabolites.

### 3.5 L‐LDH Is Responsible for the Conversion of KV Into L‐HV

Ultimately, since substantial portions of BCAAs or BCKAs were metabolized to BCHAs, the enzyme(s) responsible for their formation were investigated. Gonzalez et al. [14] already suggested that L‐LDH might be responsible for the reduction of BCKAs, even though their experiments rather demonstrate the catalysis of the reverse reaction, that is, the formation of BCKAs from BCHAs and NAD^+^. L‐LDH is known to convert the α‐KA pyruvate into L‐Lactate, while NADH serves as an electron donor, and vice versa. In this work, an in vitro assay with L‐LDH in the presence of BCKAs and NADH was performed.

**FIGURE 5:**
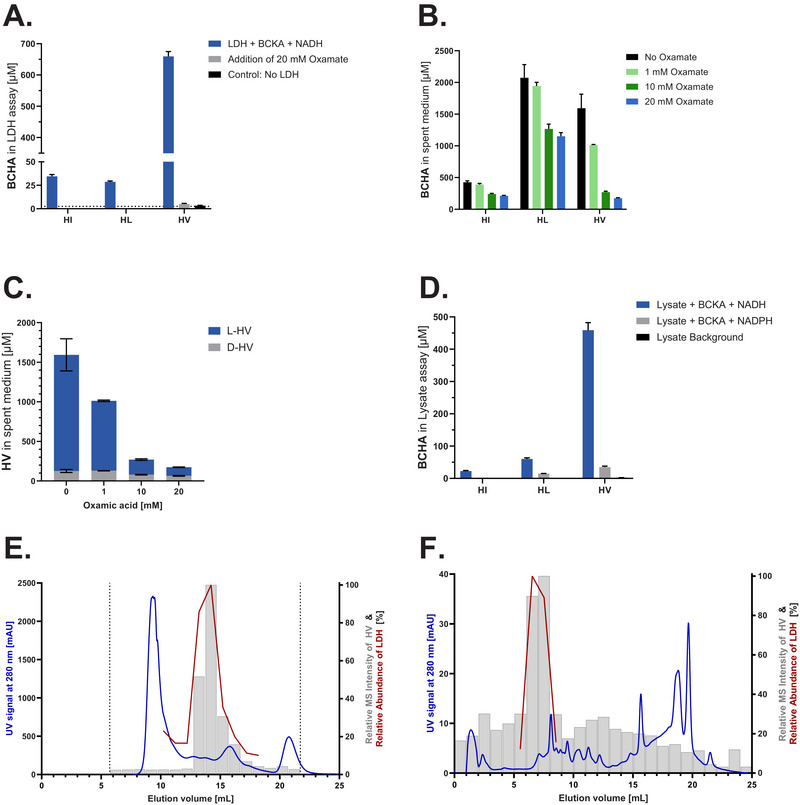
Enzymatical reduction of BCKAs forming BCHAs and impact of oxamic acid on BCHA formation. (A) Absolute concentrations of BCHAs in µM, quantified in an in vitro L‐LDH assay produced from BCKAs in the presence or absence of oxamic acid. The limit of quantitation (2.5 µM) is indicated with a horizontal, dotted line (*n* = 3). Absolute BCHA (B) and HV stereoisomer (C) concentrations in µM on day 17 of a fed‐batch cultivated with 0, 1, 10, and 20 mM oxamic acid in the basal medium in combination with equimolar replacement of Ile and Leu by KI and KL in ModiFeed Prime (*n* = 3). (D) Absolute concentrations of BCHAs in µM, formed from BCKAs and NAD(P)H in CHO lysate (limit of quantitation: 1.3 µM, *n* = 3). Two‐dimensional fractionation of CHOK1 GS lysate with (E) size exclusion chromatography followed by (F) anion‐exchange chromatography of individual fractions obtained from the first dimension. The blue line indicates the UV trace at 280 nm of the chromatographic separation. The red line indicates the relative abundance of L‐LDH, obtained by LC–MS following a tryptic digest of the collected fractions. The grey bars indicate the MS signal intensity of HV relative to the maximal value, which was determined in the catalytic assay using NADH and KV. In (E), the dotted vertical lines indicate the range of fractions, which were subjected to the catalytic assay, while in (F) all fractions were used.

Results indicate a substantial conversion of KV into HV by L‐LDH, while by far less HI and HL (5% and 4% of HV, respectively) were produced from KI and KL (Figure 5A), suggesting a better affinity of L‐LDH towards KV. In vitro, oxamic acid, a competitive L‐LDH inhibitor, reduced the formation of all three BCHAs substantially (Figure 5A) and was thus used in a fed‐batch experiment to study the involvement of the Chinese hamster L‐LDH in the formation of BCHAs. Oxamic acid was supplemented in the basal medium (0, 1, 10, and 20 mM) in combination with Cellvento ModiFeed Prime supplemented with KI/KL. Cell performance was only slightly impacted by the use of oxamic acid [data not shown], but the formation of HV was substantially decreased in a dose‐dependent manner. The addition of 1, 10, and 20 mM oxamic acid led to decreased HV formation by 36%, 83%, and 89%, respectively, indicating the involvement of L‐LDH in HV formation within CHO cells. In contrast, the concentrations of HI and HL were only reduced by maximally 50% and 44%, respectively (Figure 5B), supporting the hypothesis that different enzymes contribute to the formation of BCHAs. As this fed‐batch was not carried out with ^13^C‐labeled KI or KL, only the stereoisomer composition of HV was determined reliably. This analysis showed that the reduction of HV was mostly due to the decrease in its major portion of L‐enantiomer (2S‐conformation) (Figure 5C).

In order to evaluate the capability of lysates to form the three BCHAs, the intracellular content of CHOK1 GS cells was extracted and incubated with the three BCKAs and either NADH or NADPH. In the presence of NADH, the lysate produced mainly HV rather than HI and HL (Figure 5D). These results were surprising, considering HL was typically the predominant BCHA species in fed‐batch cultivations. Stereoisomer analysis showed that >99% of the HV was found to be the L‐enantiomer. The use of NADPH led to negligible amounts of BCHAs compared to NADH.

In order to identify the enzyme(s) mainly responsible for the formation of L‐HV, the same lysate was fractionated using a two‐dimensional chromatographic purification using SEC and AEX. A SEC column with a broad separation range was used, and the capability of the fractions to catalyze the formation of HV from KV and NADH was evaluated (Figure 5E). These fractions were further subjected to AEX (Figure 5F), where a low number of catalytically active fractions were further identified. Notably, KI and KL were also included in the catalytic assays, and little formation of their HI and HL was observed in the fractions, in clear contrast with the significant formation of HV.

To identify the enzymes of interest in the active (and their bracketing) fractions, an in‐house proteomics approach was used. Results, focusing on the relative abundance of oxidoreductases, were correlated with the activity profile. The total number of proteins identified in the SEC fractions was 1,073, while the analyzed AEX fractions contained 139. Ultimately, L‐LDH was the only identified oxidoreductase matching the profile of the catalytic assay (Figure 5E,F). The match was confirmed for multiple peptides unique to L‐LDH. Consequently, the results of the fractionation experiment suggest L‐LDH to be responsible for the formation of L‐HV. As the formation of HI and HL was low in these fractions, the identification of the enzyme responsible for their formation was not successful.

Subsequent studies on the formation of D‐HL [data not shown] focused on the overexpression of endogenous D‐2‐hydroxy acid dehydrogenases in CHOK1 GS using the CRISPRa SAM system. C‐terminal binding protein 1 (CtBP1) and 2 (CtBP2) as well as Glyoxylate/Hydroxypyruvate Reductase (GRHPR), D‐2‐Phosphoglycerate Dehydrogenase (PHGDH), FAD‐dependent D‐LDH, and D‐2‐Hydroxyglutarate Dehydrogenase (D2HDGH) were attempted to be overexpressed, yet the level of overexpression was not sufficient for meaningful assay development addressing KL reduction to D‐HL.

## 4 Discussion

In this work, BCKAs and related metabolites were investigated in CHO cultures to understand their impact on cell performance upon replacement of Ile and Leu by their respective KA sodium salts in CCF. Equimolar replacement of KI and/or KL led to a moderate decrease in cell growth, which was compensated by an increase in specific productivity, resulting in similar IgG titers at the end of cultivation. When KI and KL were supplemented in excess, cell performance decreased drastically in terms of cell growth and productivity.

The involvement of a potential limitation in the amination process (necessary to convert BCKAs into BCAAs) in the decreased cell performance upon usage of high BCKA concentrations was studied. Results confirm that none of the BCAAs (or likewise any other AA) was depleted as a result of BCKA supplementation, indicating that the amination reaction is not responsible for the decrease in cell growth and productivity. In CHO cells, cytosolic BCAT1 is the enzyme responsible for this reversible transamination and Glu is described as the natural substrate for BCKA amination, forming KG and the respective BCAAs [10, 11]. Schadewaldt et al. showed with BCAT from human skin fibroblasts that KG and Glu were the preferred substrates over other BCKAs and BCAAs for the transamination of Leu or KL, respectively, depending on the direction of the transamination [16]. However, based on experiments in CHO lysates, our group suggested previously that in case of exogenous BCKA supply, the respective other available BCAAs are used as substrates for amination rather than Glu [5], for example, amination of KL by Ile or Val forming Leu and KI or KV, respectively. The rapid increase in extra‐ and intracellular concentrations of the KAs corresponding to the donating BCAAs in this study supported this hypothesis. Importantly, the data do not exclude the additional involvement of Glu in the amination process, as the extracellular Glu levels slightly decreased in the case of equimolar replacement, while the KG levels increased compared to the positive control. Nevertheless, the changes in Glu and KG levels were less striking in the immediate response to BCKA supplementation compared to the observed increases in KA levels of donating BCAAs, particularly KV. Pereira et al. observed with CHO cells that extracellular Glu levels did not change upon disruption of BCAT1/2 expression [17], which was not anticipated based on the reduced Glu release demonstrated in glioma cells upon BCAT1 suppression [18]. Eventually, these findings also indicate that the transamination of BCAAs and BCKAs by BCAT might behave differently in CHO cells, although multiple other factors, including but not limited to growth conditions and concentrations of media components, might contribute to differing AA and KA transport and metabolism.

To assess the impact of KI and KL supplementation on cell performance and on BCAA and BCKA metabolism, the products of BCKA decarboxylation and reduction were quantified, as various metabolic intermediates and by‐products of amino acids were described recently as detrimental to CHO cells [19, 20]. In terms of BCAA‐derived metabolites, all three dcBCKAs as well as HL were previously suspected to inhibit cell growth [9, 10, 12, 21]. In the presented fed‐batch experiments, less dcBCKAs were formed per cell per day upon KI and KL supplementation in a dose‐dependent manner. These findings were unexpected, as an increased metabolization of BCKAs through the BCKDH complex was hypothesized upon exogeneous KI and KL supplementation. Among others, Paxton and Harris demonstrated that BCKAs, mostly KL, activate the BCKDH complex by inhibition of its negative regulator BCKDH kinase [22, 23]. However, the release of free dcBCKAs requires the preceding hydrolysis of the respective CoA thioesters, which was suggested to be catalyzed by acyl‐CoA thioesterases (ACOT) [14] due to their broad substrate specificity, yet this hypothesis remains unverified [24, 25, 26]. Nevertheless, this additional enzymatic step might decouple the synthesis of dcBCKAs from the BCKDH complex activity leading to the formation of CoA thioesters.

Regarding the impact of dcBCKAs on cell performance, studies of Mulukutla et al. in CHO cells demonstrate that spiking with the three individual dcBCKAs decreased cell growth in a dose‐dependent manner in the low mM range [9, 10]. Subsequent studies with BCAT1 knockout cells, which led to the suppression of endogenous dcBCKA production, revealed that reduced formation of dcKL and dcKV negatively impacts the cell‐specific productivity [11]. In more detail, the resupplementation of dcKL in these engineered cells (and dcKV to a lesser extent) increased the recombinant protein cell‐specific productivity (dcKI was not investigated). In contrast to the literature, the presented study unveils a correlation between a decreased synthesis of dcBCKAs and an increased specific productivity for conditions with equimolar replacement of Ile and Leu by their KAs. Based on the conflicting results obtained in multiple studies, dcBCKAs are unlikely to be related to either the increased specific productivity (in case of equimolar KI and KL replacement) or to the decreased cell growth observed upon KI and KL supplementation. The root cause or consequences of decreased dcBCKA formation were not studied further and are beyond the scope of this work.

Aside from dcBCKAs, the levels of BCHAs increased substantially upon KI and KL supplementation, with HV and HL reaching considerably higher concentrations than HI. Given this drastic increase, the BCHAs were hypothesized to be growth inhibitors. To investigate the impact of BCHAs on cell performance, the stereoisomeric composition of all three BCHAs was determined to create a mixture of their predominant enantiomers for supplementation during fed‐batch cultivation. Results obtained upon spiking did not differ substantially from the control in terms of cell growth and titer, leading to the conclusion that BCHAs are unlikely to be growth inhibiting in the concentration range detected upon KI and KL usage. Similar experiments were performed recently with CHO cells in batch mode [12, 14]. In both studies, BCHAs were spiked individually in the basal medium at concentrations in the low mM range. To our best understanding, racemic BCHAs were used. Consequently, the effects of the distinct stereoisomers were not considered. Furthermore, the cells were exposed to high concentrations of BCHAs already early in the growth phase, which does not reflect the conditions reported herein. Nevertheless, concentrations of 1 mM HL or 2 mM of individual BCHAs, respectively, did not change cell growth substantially [12, 14]. These concentrations fairly reflect the accumulated HL and HV levels in conditions supplemented with KI and KL in the presented fed‐batch cultivations, whereas the HI levels of 2 mM by Gonzalez et al. certainly exceeded the amounts detected in this work. Ultimately, the published results support our findings, suggesting that the level of BCHAs produced upon KL and KI feeding does not substantially inhibit cell growth.

Despite their low impact on cell growth, the formation of BCHAs was further investigated, as they represent substantial portions of supplemented BCAA or their BCKA precursors. In the literature, only little knowledge is available about their synthesis despite their prior identification in other organisms [27, 28, 29, 30, 31]. Gonzalez et al. suggested promiscuous catalysis by L‐LDH leading to the formation of BCHAs in CHO [14], which was demonstrated using L‐LDH from rabbit muscle [28] and other organisms, such as *Alcaligenes eutrophus* [31]. Results presented in this work confirm the involvement of L‐LDH in the production of BCHAs from BCKAs in the presence of NADH, however, they suggest that the reaction mainly leads to the formation of L‐enantiomers. Furthermore, results indicate that L‐LDH has a clear preference to reduce KV rather than KI and KL, which is in good agreement with the stereoisomers identified in CHO culture in the current study. This is also in alignment with the catalytic constants (*k*_cat_), previously determined for the reduction of all the three BCKAs [28]. Given the small size of L‐LDH's natural substrate pyruvate, the short length of the side‐chain is thought to be responsible for the enzyme's enhanced efficiency in reducing KV compared to KI and KL [28]. However, dedicated studies focusing on the different enzyme‐substrate interactions are required to verify this hypothesis.

High formation of HL was expected in the CHO lysate based on its concentration in conditions supplemented with KI and KL in fed‐batch. The minimal formation of HL in the CHO lysates might be due to the extraction protocol used to prepare the lysates or to the absence of activity, for example, due to the requirement of other co‐factors or coenzymes. Nevertheless, the lack of elevated HL in the lysate was considered further evidence of the involvement of other enzymes in the formation of BCHAs, particularly the D‐enantiomer of HL. While D‐HL is known to be formed by NAD‐dependent D‐2‐hydroxyisocaproate dehydrogenase (EC 1.1.1.345) and D‐LDH (EC 1.1.1.28) in bacterial strains, such as *Lactobacillus casei* [32, 33], *Lactobacillus reuteri* [34], and *Clostridium difficile* [35], D‐2‐hydroxyacid dehydrogenases are not well studied in mammalian cells [36]. Some exemplary enzymes from this family are glyoxylate/hydroxypyruvate reductase (GRHPR, EC 1.1.1.79/81), phosphoglycerate dehydrogenase (PHGDH, EC 1.1.1.95), and C‐terminal binding proteins 1 and 2 (CtBP1/CtBP2, EC 1.1.1.428). The reduction of KL and other 2‐ketocarboxylic acids [37] was demonstrated for human CtBP1; however, the capability to reduce KL or the remaining BCKAs was not shown in CHO cells. Recently, murine FAD‐dependent D‐LDH (EC 1.1.2.4), which is not related to L‐LDH and NAD‐dependent D‐LDH, was implicated in the conversion of D‐enantiomers of BCHAs into their respective KAs in the presence of FAD and Mn^2+^ [38]. Although the reverse reaction was not studied, it might play a role in the formation of D‐BCHAs from BCKAs. To advance the investigations on the aforementioned D‐2‐hydroxyacid dehydrogenases, genetic engineering using the CRISPRa SAM system was attempted. However, the level of overexpression achieved was insufficient for meaningful assays related to KL reduction. Eventually, further research is required to identify the enzyme catalyzing the reduction of KL to D‐HL.

## 5 Conclusion

In summary, this study describes the formation and impact of BCAA‐related metabolites upon the replacement of Ile and Leu by their KA derivatives in fed‐batch using a CHO expression system. The findings indicate that the decreased production of dcBCKAs as well as the substantially increased accumulation of BCHAs are not responsible for the detrimental impact of excessive KI and KL supplementation. Besides demonstrating the formation of KV as a result of KI and KL amination, our findings suggest that the reduction of the three BCKAs is likely catalyzed by multiple enzymes. L‐LDH appears to play a primary role in the formation of L‐BCHAs (2S‐conformation), mostly L‐HV, while further investigations are required to identify the enzyme(s) involved in the formation of D‐BCHAs (2R‐conformation), as this is the predominant HL enantiomer. Despite the by‐product formation, the findings highlight that media components, such as the BCKAs, can be used for next‐generation processes, if dosed appropriately.

## Author Contributions

**Philipp Reifenberg**: conceptualization, methodology, investigation, visualization, writing–original draft preparation. **Lara Rosenberger**: methodology, investigation. **Maxime Le Mignon**: methodology, investigation. **Aline Zimmer**: supervision, conceptualization, writing–reviewing and editing.

## Conflicts of Interest

Authors P.R., M.L.M., and A.Z. are employed by the company Merck Life Science KGaA. Author L.R. is employed by the company Merck KGaA.

## Supporting information




**Supporting file 1**: biot70041‐sup‐0001‐SuppMat.pdf.

## Data Availability

All data are present in the manuscript or supplementary data.
