# Supplementary material for: Replacement of Isoleucine and Leucine by their Keto Acids leads to increased formation of α‐Hydroxy Acids in Chinese Hamster Ovary cells
Source: Biotechnol J. 2025 Jun 9;20(6):e70041. doi: 10.1002/biot.70041 (PMC12149483; doi:10.1002/biot.70041)
Supplement: Supplementary file 1 — Supporting file 1: biot70041‐sup‐0001‐SuppMat.pdf. [file BIOT-20-e70041-s001.pdf]

Supporting Information

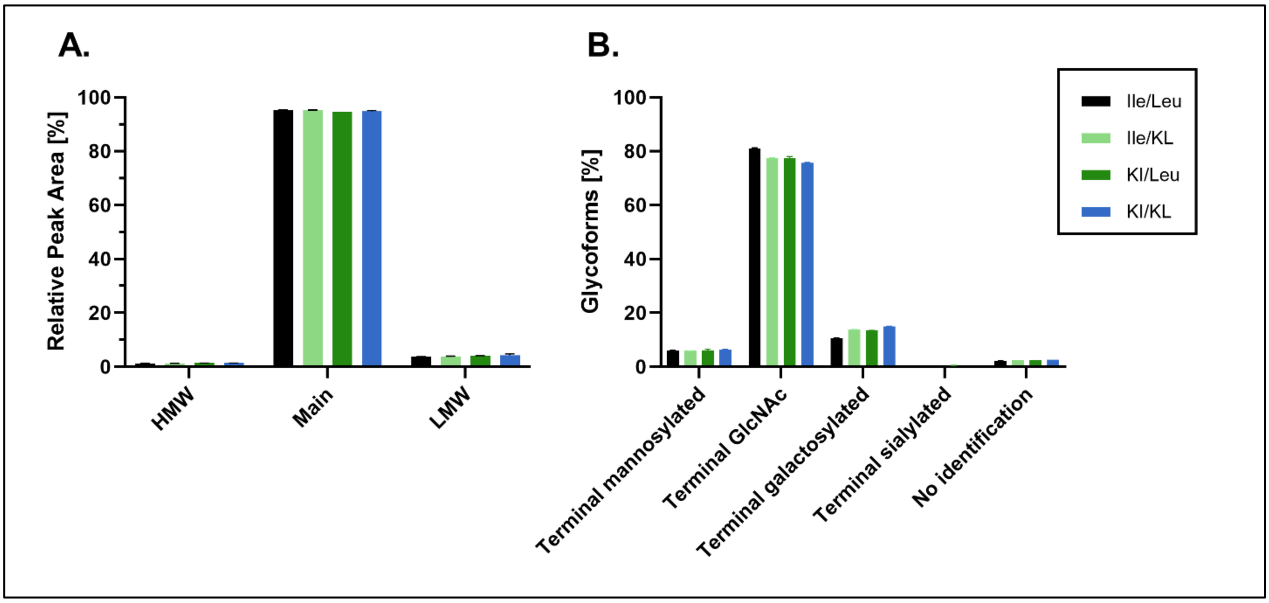

Supp. Figure 1: Critical quality attributes of the monoclonal antibody (mAb) produced by CHOK1 GS. The mAb from spent media on day 14 of the fed-batch was purified using Protein A loaded PhyTips® by PhyNexus Inc. A: Aggregation [%] was determined by use of Size Exclusion Chromatography (n=2). B: N-glycan cleavage and fluorescence labeling was performed using the "GlykoPrep®-plus with automated AssayMAP Technology" by PROzyme®. CGE-LIF was used to separate and quantify the labeled, released N-glycans (n=2).

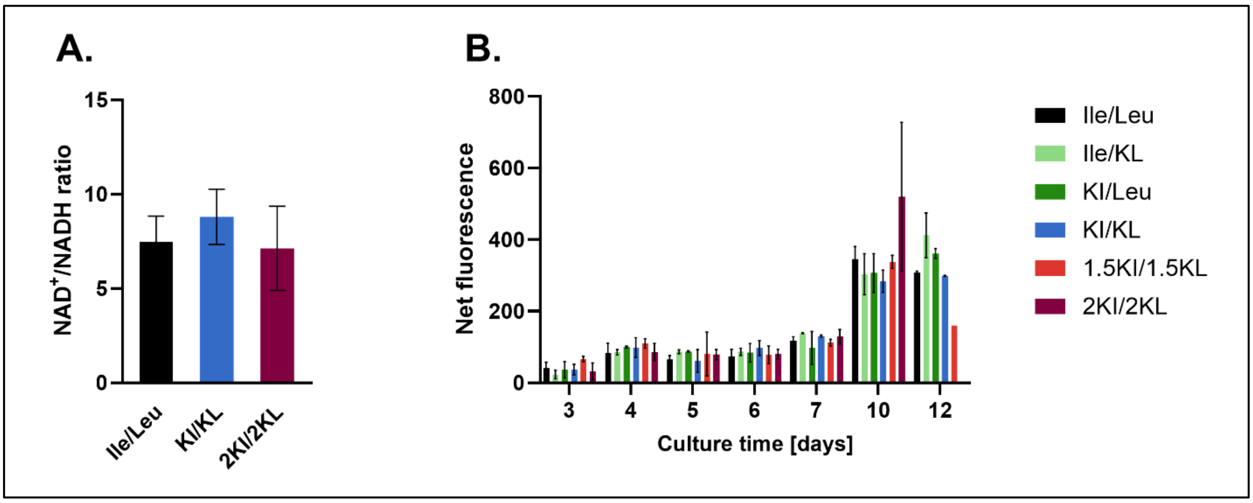

Supp. Figure 2: Assessment of metabolic and oxidative stress. A: Intracellular NAD^+/NADH ratios determined on day 7 of a fed-batch with conditions Ile/Leu, KI/KL and 2KI/2KL using a fluorometric kit (Sigma, MAK460-1KT) (n=4). B: Intracellular reactive oxygen species (ROS) levels, represented by the net fluorescence, were assessed on several days of a fed-batch using Carboxy-H_2DCFDA as fluorescent indicator for ROS (n=2).

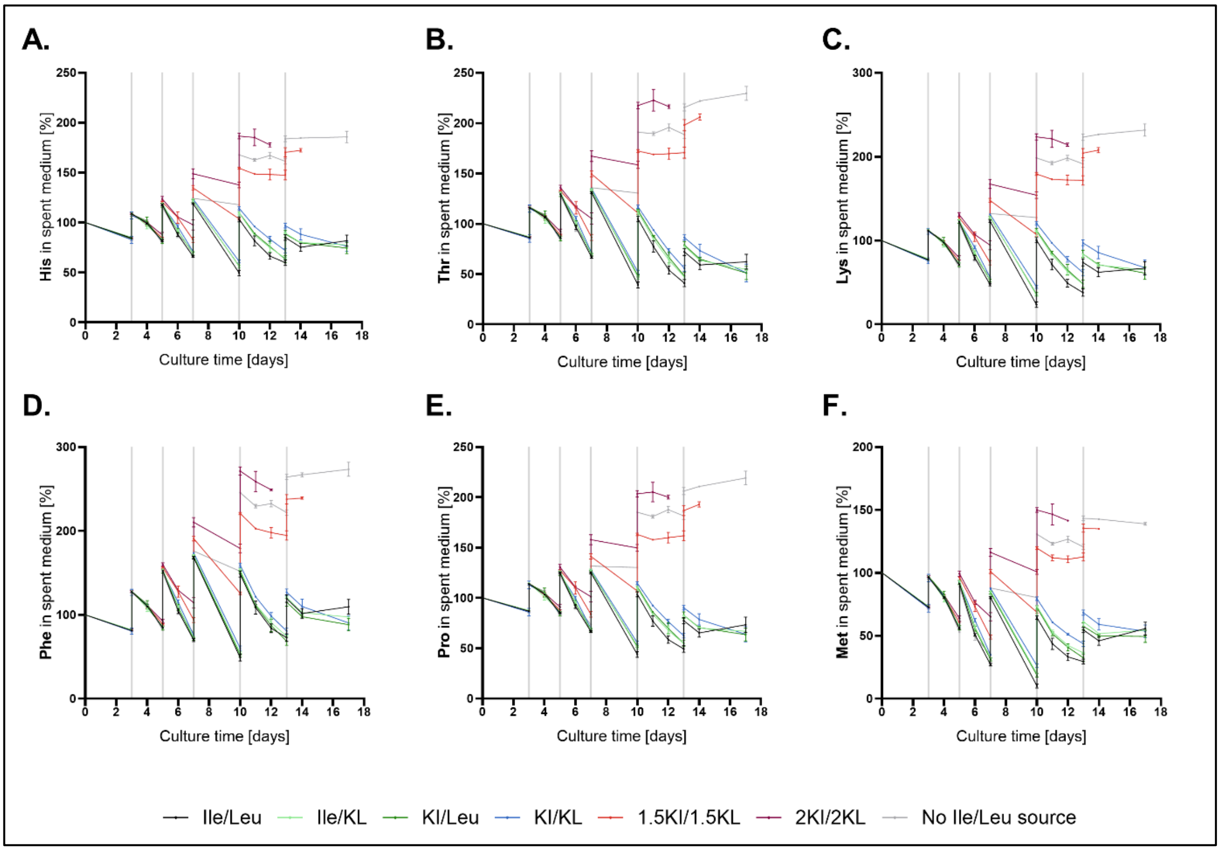

Supp. Figure 3: Extracellular levels of essential amino acids Histidine (His), Threonine (Thr), Lysine (Lys), Phenylalanine (Phe), Proline (Pro) and Methionine (Met) obtained in fed-batch with different combinations of branched-chain keto and amino acids. Concentrations were determined by UPLC-UV following AccQ-Tag derivatization. A-F: Essential amino acids in spent medium normalized to their starting concentration (n=2). Grey vertical lines indicate feed supplementation.

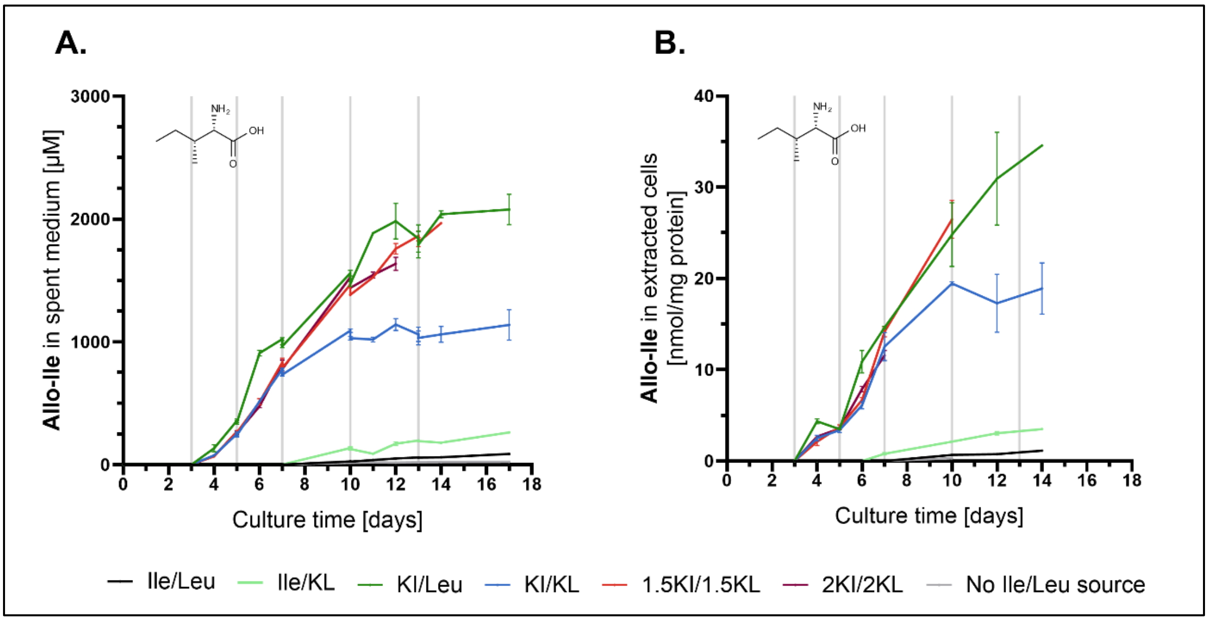

Supp. Figure 4: Extra- and intracellular levels of Allo-Ile obtained in fed-batch with different combinations of keto and amino acids. Concentrations were determined by UPLC-UV following AccQ-Tag derivatization. A: Allo-Ile in spent medium in µM (n=2). B: Amount of intracellular Allo-Ile normalized to the total protein content in nmol/mg protein (n=2). Grey vertical lines indicate feed supplementation.

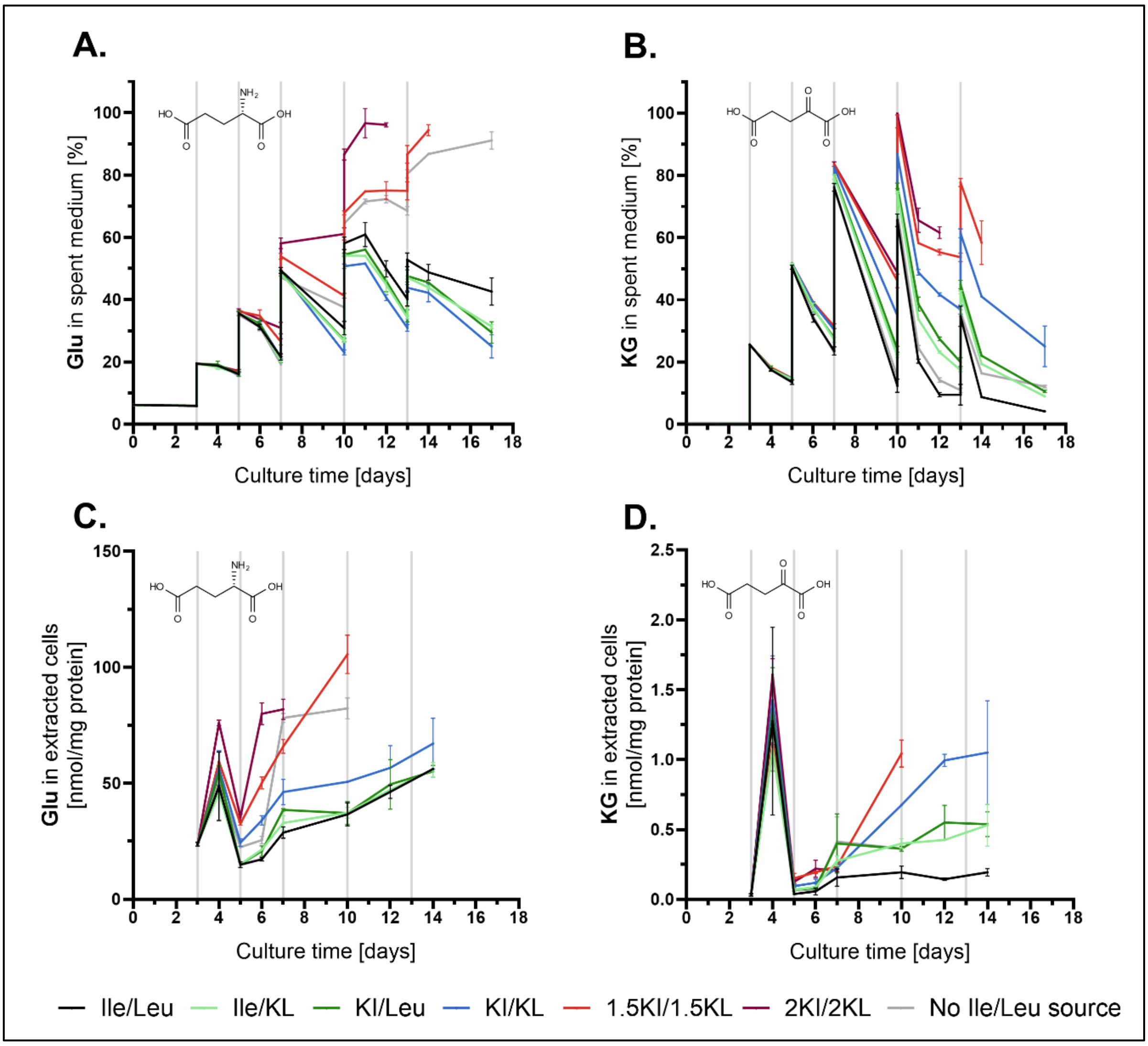

Supp. Figure 5: Extra- and intracellular levels of glutamate (Glu) and α-ketoglutarate (KG) obtained in fed-batch with different combinations of keto and amino acids. Glu concentrations were determined by UPLC-UV following AccQ-Tag derivatization and KG was quantified by LC-MS/MS after O-BHA derivatization. A: Extracellular Glu normalized to the maximal concentration [%] (n=2). B: Extracellular KG concentration normalized to the maximal concentration [%] (n=2). C: Intracellular Glu content normalized to the total protein content in nmol/mg protein (n=2). D: Intracellular KG content normalized to the total protein content in nmol/mg protein (n=2). Grey vertical lines indicate feed supplementation.

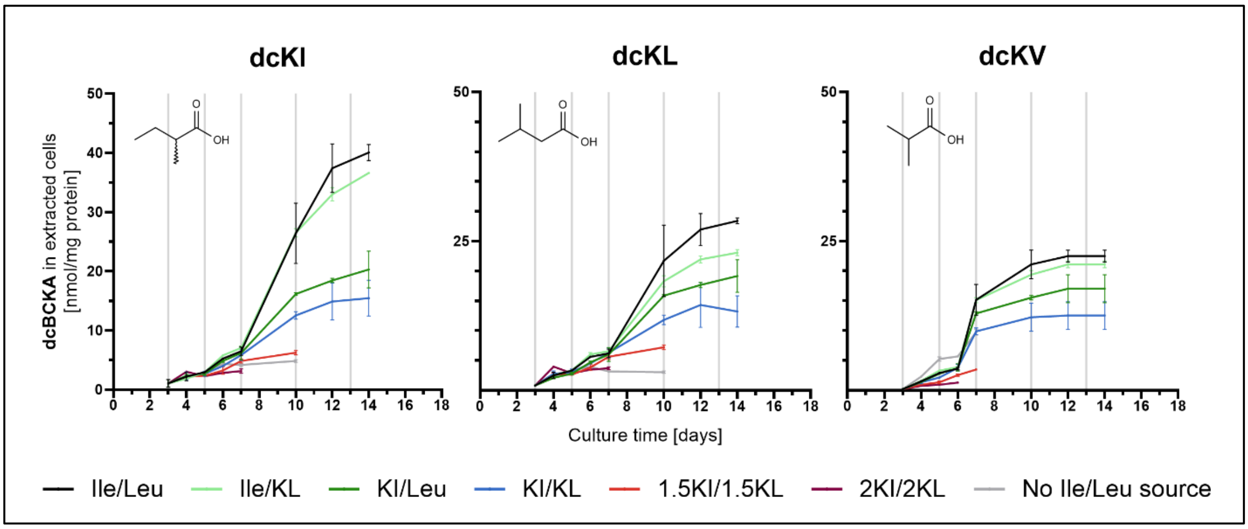

Supp. Figure 6: Intracellular levels of the BCKAs' decarboxylation products obtained in fed-batch with different combinations of keto and amino acids normalized to the total protein (TP) content in nmol/mg protein (n=2). Concentrations were determined by LC-MS/MS upon O-BHA derivatization. Grey vertical lines indicate feed supplementation.

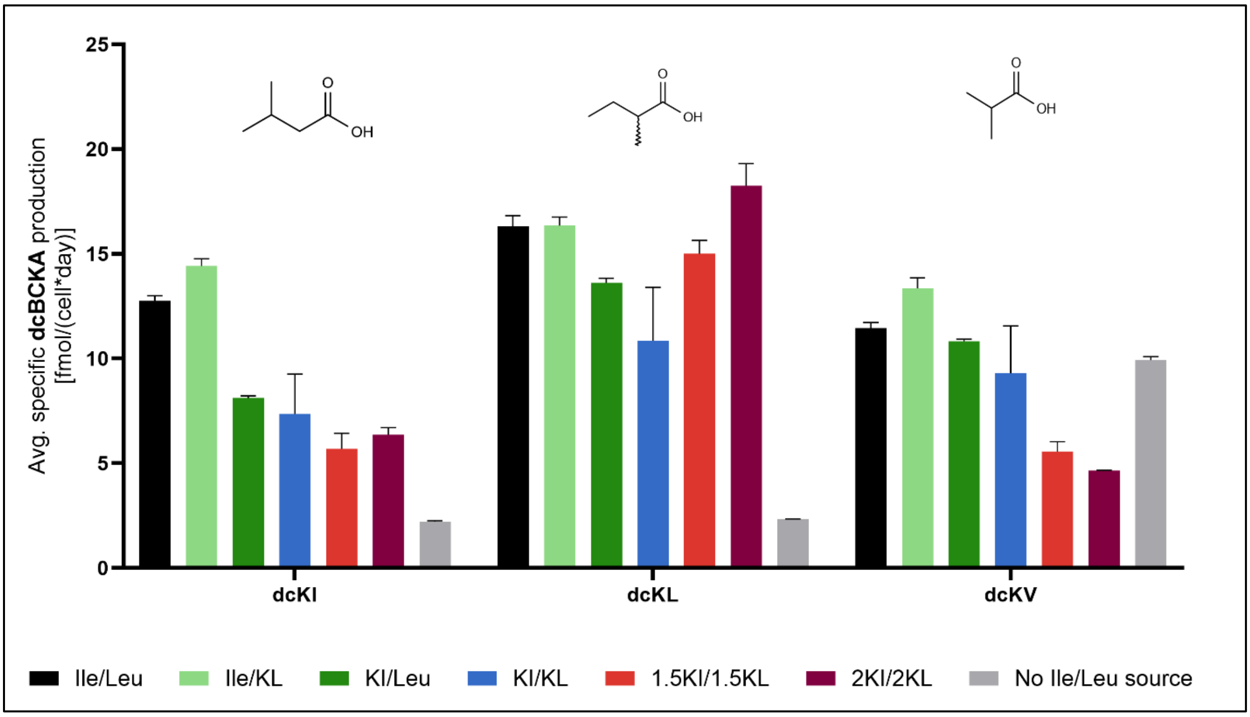

Supp. Figure 7: Average specific dcBCKA production in fmol/(cell*day) determined by dividing the dcBCKA concentration at the end of cultivation by the integral of viable cells (IVC) (n=2).

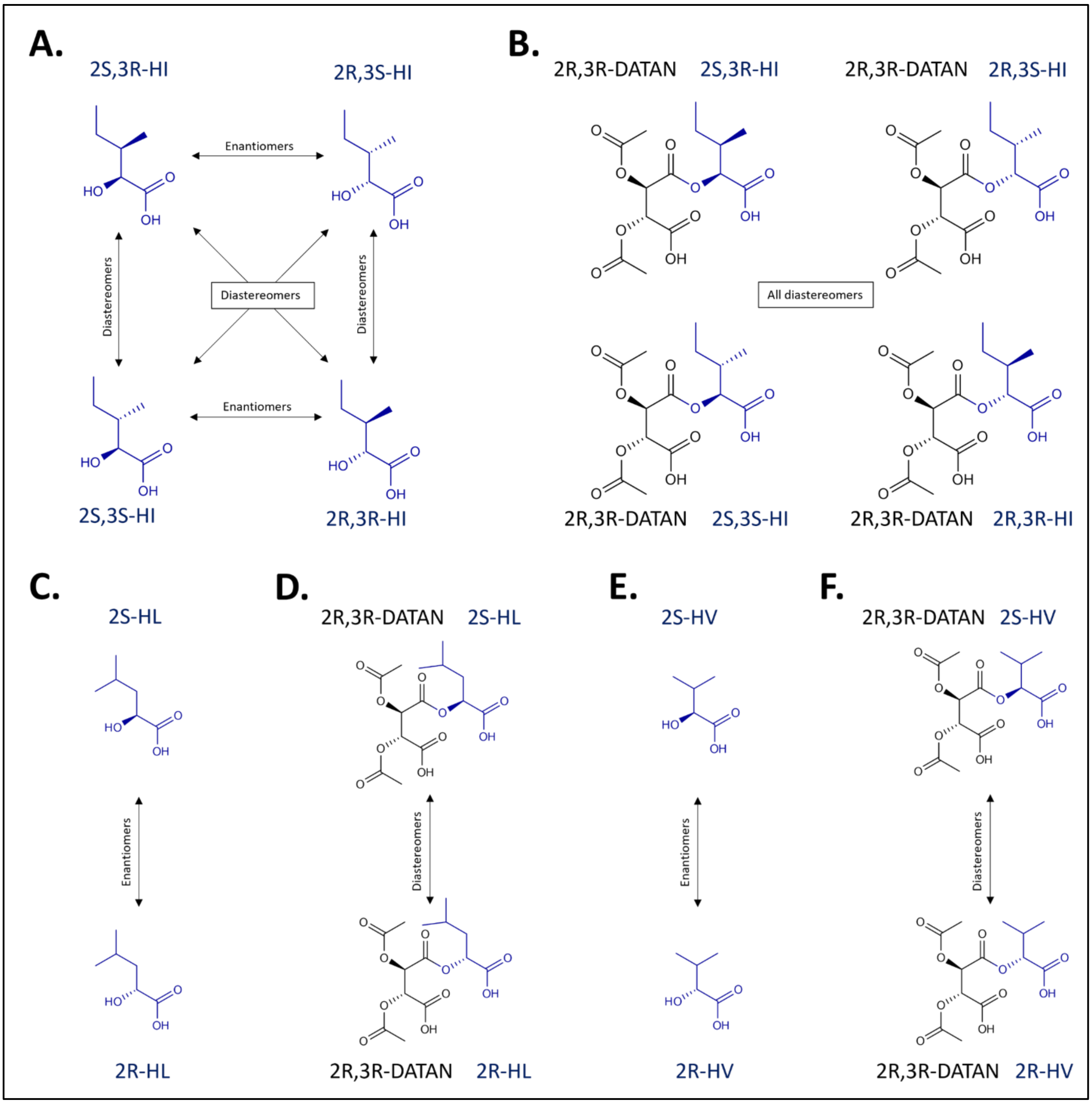

Supp. Figure 8: Stereoisomers of 2-hydroxyisoleucine (HI), 2-hydroxyleucine (HL) and 2-hydroxyvaline (HV) and their respective esters upon derivatization with (+)-O.O'-diacetyl tartaric acid anhydride ((2R,3R)-DATAN). A: HI diastereomers/enantiomers. B: DATAN-Derivatized HI diastereomers. C: HL enantiomers. D: DATAN-derivatized HL diastereomers. E: HV enantiomers. F: DATAN-derivatized HV-diastereomers.

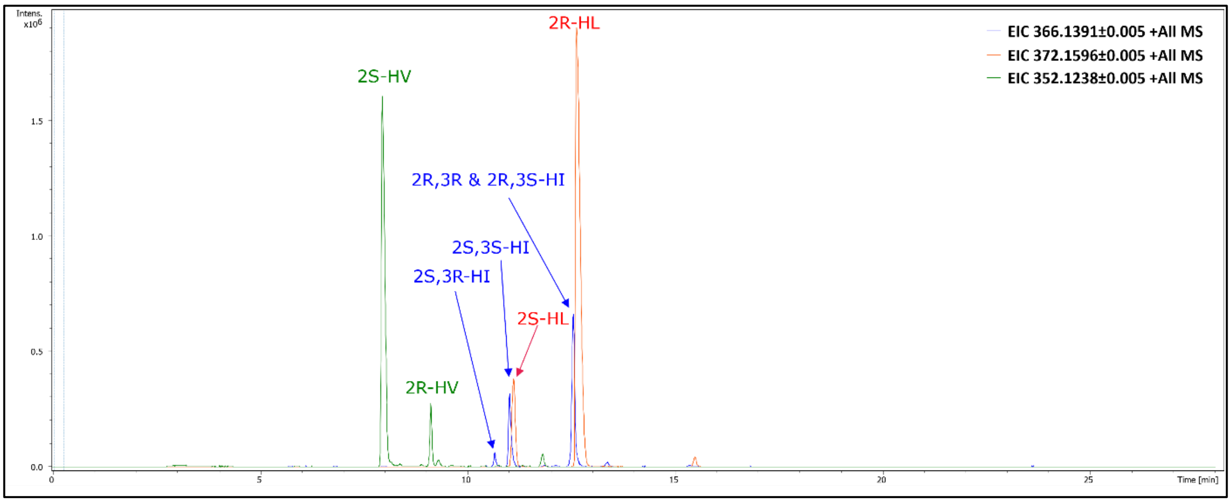

Supp. Figure 9: Extracted ion chromatogram of the reversed-phase separation of BCHA stereoisomers represented by an extracellular sample of the condition supplemented with KI and KL on day 17, derivatized with the (+)-DATAN. Use of ^{13}C-labeled Leu and KL allowed for differentiation between HI (blue) and HL (red) isomers. The extracted ion chromatogram of HV is represented in green.

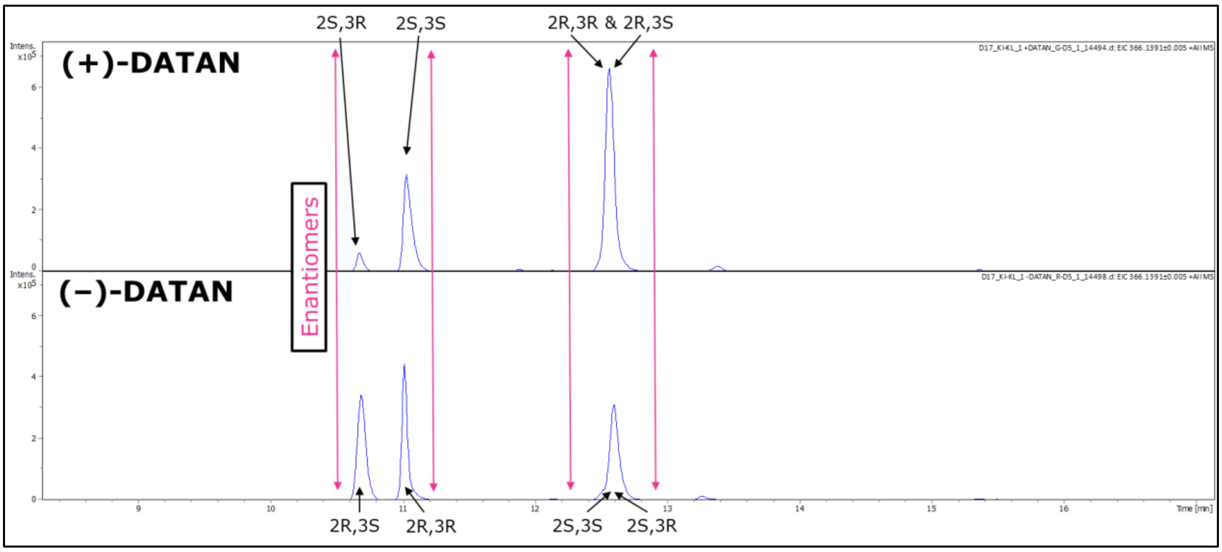

Supp. Figure 10: Extracted ion chromatograms of the reversed-phase separation of HI diastereomers represented by an extracellular sample of the condition supplemented with KI and KL on day 17, derivatized with the (+)-DATAN (top) and (–)-DATAN (bottom). The parallel use of both enantiomers of the derivatization reagent allowed for calculation of all four HI diastereomers by individual determination of resolved species. As an example, the ratio of 2S,3R to 2S,3S to the sum of 2R,3R and 2R,3S can be determined from the sample derivatized with (+)-DATAN. In order to calculate the fraction of all four diastereomers, the ratio of 2R,3R and 2R,3S can be obtained from (–)-DATAN derivatization.

Supp. Table 1: LC-MS conditions for analysis of branched-chain hydroxy acids

**LC-MS instrument**	1290 Infinity II instrument coupled to an G6495C triple quadrupole mass spectrometer (Agilent)
**Column**	Acquity UPLC HSS T3; 150 x 2.1 mm, 1.8 µm (Waters)
**Column temperature [°C]**	40
**Mobile phase**	Water + 20 mM ammonium formate + 0.1% formic acid (**A**), Methanol (**B**)

**Gradient**	Time [min]	**A** [%]	**B** [%]
	0.0	95.0	5.0
	2.0	95.0	5.0
	4.0	80.0	20.0
	6.0	70.0	30.0
	14.0	60.0	40.0
	14.5	0.0	100.0
	16.5	0.0	100.0
	16.6	95.0	5.0
	18.0	95.0	5.0

**Flow rate [ml/min]**	0.30
**Injection volume [µl]**	2.0
**Acquisition & data evaluation software**	MassHunter Workstation: LC/MS Data acquisition and Quantitative Analysis for QQQ Version 10.1 (Agilent)
**Ionization mode (polarity)**	ESI (negative)
**Acquisition mode**	Multiple Reaction Monitoring
**Gas temperature [°C]**	250
**Gas flow [L/min]**	11
**Nebulizer [psi]**	25
**Sheath gas temperature [°C]**	250
**Sheath gas flow [L/min]**	11
**Capillary voltage (negative) [V]**	3000
**Nozzle voltage (negative) [V]**	1500
**High Pressure RF (negative) [V]**	150
**Low Pressure RF (negative) [V]**	60

Multiple Reaction Monitoring transitions for native and ISTD analytes:

**Analyte**	**Compound**	**Precursor ion [m/z]**	**Product ion [m/z]**	**CE [V]**
1	2-Hydroxy-3-methylbutyric acid	117.0	71.2	12
	2-Hydroxy-3-methylbutyric acid-d_7	124.0	77.2	12
2	2-Hydroxy-3-methylpentanoic acid	131.0	85.1	12
	2-Hydroxy-3-methylpentanoic acid-d_{10}	141.0	94.2	12
3	2-Hydroxy-4-methylpentanoic acid	131.0	85.1	12
	2-Hydroxy-4-methylpentanoic acid-d_3	134.0	88.2	12

Supp. Table 2: LC-MS conditions for analysis of branched-chain keto acids, their decarboxylation products and ketoglutarate

**LC-MS instrument**	1290 Infinity II instrument coupled to an G6495C triple quadrupole mass spectrometer (Agilent)		
**Column**	Luna Omega Polar C18 column, 100 x 2.1 mm, 1.6 µm, 100 Å<br><br>(Phenomenex)		
**Column temperature [°C]**	45		
**Mobile phase**	Water + 0.1% formic acid (**A**),<br>Methanol (**B**)		
**Gradient**	Time [min]	**A** [%]	**B** [%]
	0.0	90.0	10.0
	0.5	90.0	10.0
	5.0	45.0	55.0
	7.5	35.0	65.0
	8.0	15.0	85.0
	9.5	15.0	85.0
	9.6	5.0	95.0
	11.0	5.0	95.0
	11.3	90.0	10.0
	12.3	90.0	10.0
**Flow rate [ml/min]**	0.50		
**Injection volume [µl]**	5.0		
**Acquisition & data evaluation software**	MassHunter Workstation: Data acquisition and Quantitative Analysis for QQQ Version 10.1<br>(Agilent)		
**Ionization mode (polarity)**	ESI (positive)		
**Acquisition mode**	Multiple Reaction Monitoring		
**Gas temperature [°C]**	250		
**Gas flow [L/min]**	11		
**Nebulizer [psi]**	25		
**Sheath gas temperature [°C]**	250		
**Sheath gas flow [L/min]**	11		
**Capillary voltage (negative) [V]**	3000		
**Nozzle voltage (negative) [V]**	1500		
**High Pressure RF (negative) [V]**	150		
**Low Pressure RF (negative) [V]**	60		

Multiple Reaction Monitoring transitions for native and ISTD analytes:

**Analyte**	**Compound**	**Precursor ion [m/z]**	**Product ion [m/z]**	**CE [V]**
1	2-Methylpropionic acid	194.0	91.1	19
	2-Methylpropionic acid-d_7	201.0	91.1	19
2	2-Methylbutyric acid	208.0	91.1	16
	2-Methylbutyric acid-d_3	211.0	91.1	28

3	3-Methylbutyric acid	208.0	91.1	20
	3-Methylbutyric acid-d_9	217.0	91.1	20
4	3-Methyl-2-oxobutyric acid	327.0	91.1	20
	3-Methyl-2-oxobutyric acid-$^{13}C_5$	332.0	91.1	20
5	2-Ketoglutaric acid	462.0	91.1	16
	2-Ketoglutaric acid-$^{13}C_4$	466.0	91.1	16
6	3-Methyl-2-oxopentanoic acid	341.0	91.1	16
	3-Methyl-2-oxopentanoic acid-d_8	349.0	91.1	20
7	4-Methyl-2-oxopentanoic acid	341.0	91.1	64
	4-Methyl-2-oxopentanoic acid-d_3	344.0	91.1	24

Supp. Methods 1: (A) Protocol for tryptic digest of SEC and AEX fractions. (B) LC-MS parameters for peptide quantitation of tryptic digests.

(A) 50 µL of sample were mixed with 5 µL 1 M tris-HCl at pH 8 and then denatured and reduced by addition of each 1 µL of ProteaseMAX (in 50 mM $(NH_4)_2CO_3$) (Promega) and 250 mM DTT (Thermo Fisher Scientific) followed by an incubation at 56°C for 60 min with 600 rpm. Consequently, free cysteines were alkylated by addition of 2 µL of 250 mM iodoacetamide (Thermo Fisher Scientific). After light-protected incubation at room temperature for 45 min, excess of iodoacetamide was quenched by adding 1 µL of 250 mM DTT. Then, 35 µL of 100 mM tris-HCl at pH 8, 1 µL of 1 M $CaCl_2$ (Merck KGaA) and 1 µL of trypsin (Roche) at 1 µg/µL (in 50 mM acetic acid) were successively added. After an incubation at 37°C for 17 h, enzymatic digestion was stopped by addition of 1 µL formic acid and incubation for 10 min at 37°C with 600 rpm. Ultimately, the samples were centrifuged at 14000 rcf for 15 min to remove degraded surfactant and 50 µL of supernatant were transferred in a LC vial containing 50 µL of 4% ACN / 0.2% Trifluoracetic acid.

(B) Samples were separated using a nanoRSLC3000 (Thermo Fisher Scientific, San Jose, CA, United States) coupled to an ESI-Q-ToF mass spectrometer (Impact II, Bruker Daltonics, Bremen, Germany) equipped with a CaptiveSpray source (Bruker Daltonics, Bremen, Germany). 8 µL of sample were loaded in 1% ACN in water with 0.1% trifluoracetic acid onto an Acclaim PepMap trap column (100 µm x 20 mm, Thermo Fisher Scientific) at 5 µL/min. The separation was carried out on a nanoEase M/Z Peptide CSH C18 (130 Å, 1.7 µm, 75 µm x 250 mm, Waters) with 300 nL/min at 40°C. A two-eluent gradient with 0.1% formic acid in water (eluent A) and 80% ACN in water with 0.1% formic acid (eluent B) was applied as follows (min/%B): 0/5, 5/5, 42/40, 43/60, 45/95, 50/95, 60/5. The MS acquisition was conducted in positive mode, with a capillary voltage of 1600 V. The dry gas (150°C) was set to 3.0 L/min. MS spectra were obtained in the m/z range of 150–2200 at a scan rate of 2 Hz. The MS-MS/MS cycle lasted 3 s and the MS/MS scan level was set to 32 Hz and 8 Hz for signals above 25000 and 2500 counts, respectively.
